# Combined influence of polymeric and mineral fibres on fresh-state performance and fracture properties of high-performance self-compacting concrete

**DOI:** 10.1038/s41598-026-41949-7

**Published:** 2026-03-10

**Authors:** Piotr Smarzewski, Krystian Błaszczyk

**Affiliations:** 1 Faculty of Civil Engineering and Geodesy, Military University of Technology, 2 Gen. Sylwestra Kaliskiego, Warsaw, 00-908 Poland; 2 Doctoral School, Faculty of Civil Engineering and Geodesy, Military University of Technology, 2 Gen. Sylwestra Kaliskiego, Warsaw, 00-908 Poland

**Keywords:** High-performance self-compacting concrete, Hybrid fibres, Fresh properties, Mechanical properties, Fracture parameters, Engineering, Materials science

## Abstract

This study investigates the influence of polyolefin, polypropylene (PP), basalt, and polyvinyl alcohol (PVA) fibres, applied individually and in hybrid systems, on the rheology-related fresh-state behaviour and fracture performance of high-performance self-compacting concrete (HPSCC). Nine concrete mixtures were formulated, including a fibre-free reference and eight reinforced variants with short (12 mm) and long (38 mm) fibres. Fresh-state properties were evaluated using V-funnel, L-box and J-ring tests, while hardened specimens were evaluated for compressive, tensile and flexural strength, as well as fracture energy and crack propagation characteristics. The results demonstrate that the fibre type and geometry significantly affect both workability and mechanical performance. Crimped PP fibres (38 mm) achieved the highest compressive strength (+ 19.9%) and tensile strength (+ 64.8%), while short PVA fibres provided the greatest improvement in flexural strength (+ 122%). The highest fracture energy (~ 1879 J/m²) and post-cracking ductility were obtained for hybrid systems combining long ductile and short stiff fibres (PD38 + PVA12), confirming the effectiveness of multiscale reinforcement. However, enhanced fracture resistance often came at the expense of workability, especially in PVA-rich systems. Future research should address this balance by optimising hybrid ratios and admixture design. The presented dataset provides reproducible results linking fresh-state behaviour with fracture performance and offers guidance for the design of fibre-reinforced HPSCC balancing self-compactability and mechanical resilience.

## Introduction

Concrete is one of the most widely used construction materials worldwide. In 2020 alone, global cement production was estimated at approximately 4.17 billion tonnes^1^. Alongside steel, timber, and ceramic-based products, concrete remains a fundamental structural material, primarily due to its relatively low production cost, availability of raw materials, and ease of use. A key characteristic of concrete is its significantly higher compressive strength compared to tensile strength – often with a ratio of up to 10:1, depending on the mix design. This inherent disparity increases the risk of cracking, which can lead to a reduction in load capacity and, ultimately, structural failure^2^.

To mitigate this drawback, conventional steel reinforcement is typically used, its amount and distribution being tailored to specific loading conditions. However, increasing attention is being paid to the use of dispersed fibres as admixtures in concrete, which can reduce the dependency on traditional steel reinforcement, thus reducing construction costs and accelerating project execution^3^. Furthermore, the adoption of self-compacting concrete (SCC) eliminates the need for mechanical vibration, which is particularly advantageous in densely reinforced or complex-shaped structures^4^.

The incorporation of fibres improves the mechanical performance of concrete, improves both compressive and flexural strength, and increases resistance to brittle failure^5^. Fibre reinforcement can effectively control crack widths, reduce brittleness, and enhance the energy absorption capacity of the composite^6,7^. Fibres also improve the deformability and resistance to fracture of the material. As shown by Branston et al.^8^, basalt fibres have a more pronounced effect on crack resistance than polypropylene fibres. The effectiveness of fibres depends not only on their material type, but also on their length, diameter, shape, and bonding capacity with the cement matrix^9–11^. The proper design of fibre-reinforced mixes requires careful selection of fibre properties to optimise concrete performance^12–14^. Recent reviews have also highlighted that the synergistic effects of hybrid fibres can significantly improve both mechanical and durability performance of high-performance concretes when optimally proportioned^15^.

Due to the risk of corrosion and the increased self-weight associated with steel fibres, synthetic alternatives are increasingly being used. These non-metallic fibres offer numerous advantages: they have a lower carbon footprint, are corrosion-resistant, and in the case of fire exposure, polypropylene fibres melt, thereby relieving internal vapour pressure and reducing the risk of explosive spalling. Additionally, polymer fibres are typically less expensive than steel fibres. However, it should be noted that polypropylene fibres have a relatively low elastic modulus, which limits their ability to carry tensile loads, an effect that can be counterbalanced by combining them with stiffer fibres such as basalt^16,17^.

According to Gołaszewski^18^, self-compacting concrete has the ability to flow under its own weight, completely filling the formwork and encapsulating the reinforcement without the need for mechanical consolidation. The fresh properties of SCC are characterised by its viscosity, flowability, and resistance to segregation. The combination of SCC with fibre reinforcement allows the design of slender structural elements while maintaining both mechanical performance and fresh-state workability.

Steel fibres remain the most commonly used reinforcement, typically 25 to 60 mm in length and represent 0.5 to 2.0% of the concrete volume^19–21^. Even small amounts reduce shrinkage and enhance mechanical performance; however, they may adversely affect workability, especially when fibre lengths exceed 40 mm. To counteract this, various admixtures are used to improve flowability, reduce cement demand, and tailor rheological properties to specific requirements. As shown by Feng et al.^22^, the addition of steel fibres improved both compressive and flexural strength, while polypropylene and polyvinyl alcohol (PVA) fibres improved flexural performance. The influence of glass, basalt and polypropylene fibres on the rheological and mechanical properties of concrete has also been confirmed by^23–25^. Furthermore, Liu et al.^26^ demonstrated the synergistic improvement achieved by combining steel and polypropylene fibres. In light of the increasing environmental demands, the incorporation of waste materials into concrete production is seen as a promising direction. For example, recycled vehicle tyre fibres^27^ and natural plant-based fibres^28^ have shown favourable effects on concrete strength and workability.

Despite the extensive body of research on fibre-reinforced concretes, existing studies on hybrid fibre systems are often limited to selected mechanical properties, focus predominantly on metallic fibres, or are conducted using different concrete matrices, which hinders direct comparison of results. Moreover, the combined influence of fibre type, length and hybridisation on both fresh-state performance and fracture-related behaviour is rarely assessed within a single, internally consistent experimental framework. This limitation is particularly critical for high-performance self-compacting concrete (HPSCC), where maintaining self-compactability while enhancing post-cracking resistance represents a major design challenge.

In response to these research gaps, the present study systematically investigates basalt, polymeric and polyolefin fibres of two distinct lengths (12 mm and 38 mm), used individually and in hybrid configurations, within a unified HPSCC matrix. The study simultaneously evaluates fresh-state performance, strength, fracture energy and crack evolution, allowing the quantification of the trade-off between self-compactability and fracture resistance. By combining fresh-state qualification tests with fracture-energy-based metrics, crack opening and displacement analysis, this work provides an internally consistent and reproducible experimental dataset that extends existing knowledge on multiscale fibre reinforcement and offers practical guidance for the mix design and application of fibre-reinforced HPSCC.

## Experimental programme

The experimental investigation involved the preparation and testing of nine different high-performance self-compacting concrete (HPSCC) mixtures, including a control mix and eight variants reinforced with various types of fibres. The compositions were designed to ensure not only adequate self-compacting properties but also compressive strengths that exceed 60 MPa after 28 days of curing, qualifying the materials as high-performance concrete (HPC). Special emphasis was placed on the precise characterisation of each fresh mixture before casting, according to established procedures for rheological evaluation.

### Materials, mixture proportions and preparation of specimens

The main component of the binder was Portland cement CEM I 52.5 R, which met the criteria of EN 197-1^29^. This cement is characterised by rapid strength development and high fineness, with a specific surface area of 4221 cm^2^/g and a compressive strength at 28 days of 65.5 MPa. The complete physical and chemical parameters are presented in Table 1.

**Table 1:** Properties of portland cement CEM I 52.5 R.

Parameter	Unit	Value	Requirement	Standard
Initial setting time	min	163	> 45	^30^
Final setting time	min	199	—	
Soundness	mm	1.2	≤ 10.0	
Blaine-specific surface area	cm^2^/g	4221	—	^31^
Specific gravity	g/cm^3^	2.54	—	
SO₃ content	%	3.01	≤ 4.0	^32^
Cl⁻ content	%	0.061	≤ 0.1	
Alkali content (Na₂Oeq)	%	0.57	≤ 0.6	
Insoluble residue	%	0.65	≤ 5.0	
Loss on ignition	%	2.91	≤ 5.0	
Compressive strength 2 days 28 days	MPa	34.5 65.5	≥ 30.0 ≥ 52.5	^33^

The binder system was supplemented with siliceous fly ash to improve particle packing and durability, through its pozzolanic activity, as well as with metakaolin (MK-40), which contributes to pore refinement and strength enhancement. An additional siliceous component, a mineral additive based on zeolite Z-50, was introduced to accelerate the performance of early age and reduce the porosity of the composite structure.

Quartz sand with a maximum particle size of 0.5 mm served as the only aggregate, ensuring a dense packing of fine particles and compatibility with SCC technology. This choice was aimed at achieving a dense granular skeleton and minimising the effect of the interfacial transition zone, which is especially important in fibre-reinforced matrices. Consequently, the obtained results should be interpreted in the context of fine-grained, sand-based HPSCC matrices, in which the role of fibres can be assessed without the additional variability introduced by coarse aggregate. Future studies should verify whether the observed trends remain valid for conventional SCC incorporating coarse aggregate, where fibre–aggregate interactions may further influence crack development and workability.

To ensure self-compacting behaviour in the presence of fibres, a series of advanced chemical admixtures were introduced: ISOSIL 800 (colloidal nanosilica), ISOFLOW 825 (superplasticizer), ISOPLAST 1243 (viscosity modifier), and ISODENSE (high-density dispersant). These admixtures were carefully dosed to provide high fluidity, cohesion and segregation resistance. The selection and dosage of chemical admixtures were governed by the need to ensure stable self-compacting behaviour across all fibre-reinforced mixtures, rather than by an optimisation of individual admixture effects. A constant admixture system was intentionally adopted for all mixtures to isolate the influence of fibre type, length and hybridisation on fresh-state and fracture-related performance. The superplasticizer was dosed to compensate for the high binder content and low water-to-binder ratio typical of HPSCC, while the viscosity-modifying admixture was introduced to counteract potential segregation and fibre-induced blocking effects. Colloidal nanosilica and the dispersant were incorporated to enhance particle packing density, matrix cohesion and fibre–matrix interaction, which is particularly critical in fibre-rich self-compacting systems. The applied dosages were established based on preliminary trial mixes and previous experience with fibre-reinforced SCC reported in the literature, ensuring consistent flowability within EFNARC limits while maintaining mechanical integrity.

**Fig. 1:**
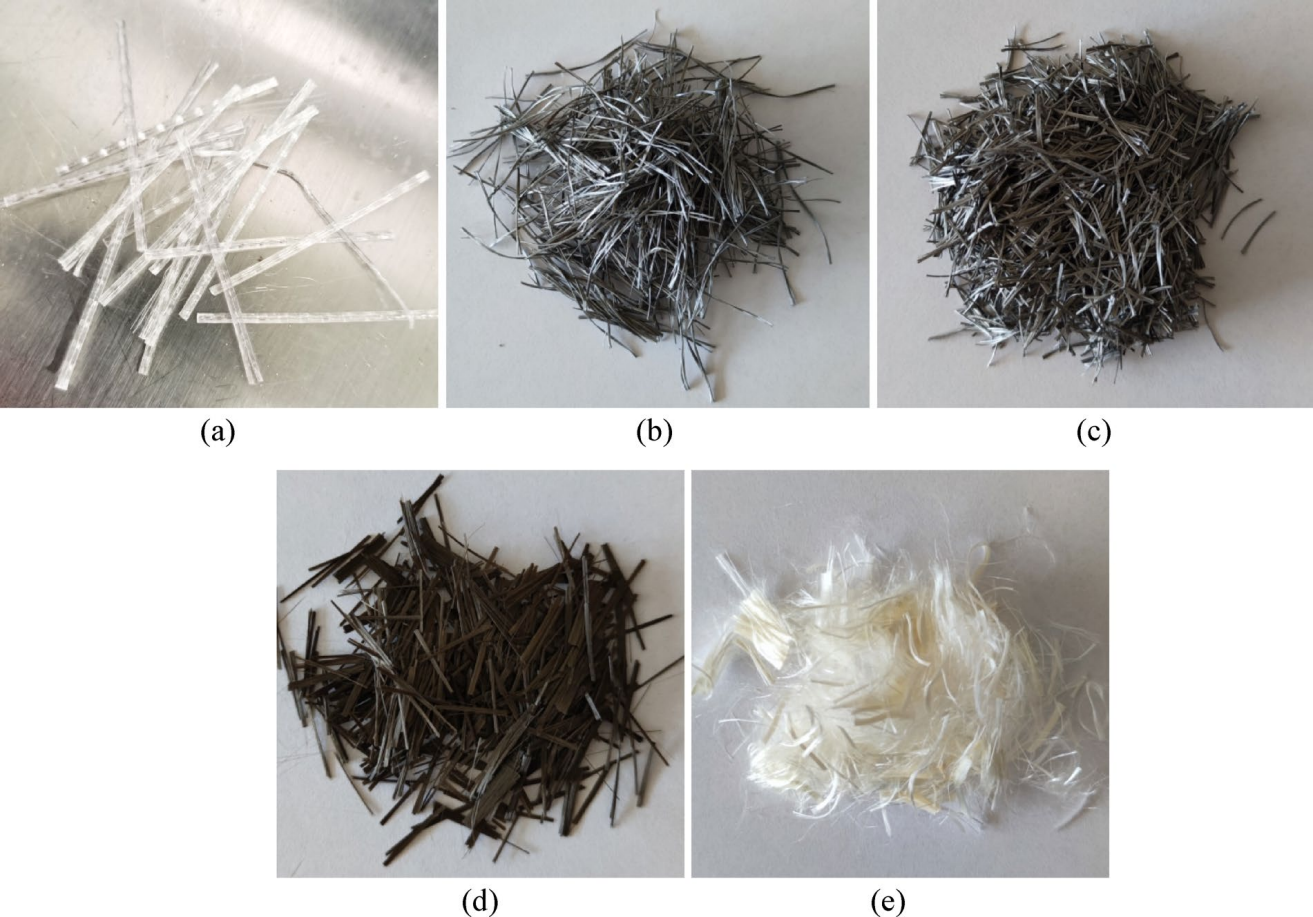
(**a**) Polyex Duro 38, (**b**) Polyex Mesh 38, (**c**) Polyex Mesh 12, (**d**) Basalt fibres 12 and (**e**) Polyvinyl alcohol fibres.

Depending on the variant, the mixtures were reinforced with synthetic or mineral fibres of various origins. Polypropylene fibres (Polyex Duro, 38 mm in length) were chosen for their balance between tensile strength (~ 600 MPa) and thermal decomposition properties, which increase fire resistance (Fig. 1a). Polyolefin fibres (Polyex Mesh 2000, 38 mm and 12 mm in Fig. 1b, c) offered corrosion resistance and stable mechanical performance, while basalt fibres (12 mm) presented in Fig. 1d, provided high stiffness (modulus up to 110 GPa) and excellent thermal stability. Additionally, short polyvinyl alcohol (PVA) fibres (12 mm) with high tensile strength (more than 1650 MPa) were incorporated due to their remarkable ability to bridge microcracks and improve ductility (see Fig. 1e).

The selection of fibre types and lengths was guided by a multiscale reinforcement concept, in which long macrofibres (38 mm) were intended to bridge macrocracks and enhance post-cracking ductility, while short microfibres (12 mm) were used to control microcrack initiation and early crack propagation. In several mixes, a hybrid fibre approach was employed by combining short and long fibres to obtain a multiscale reinforcement effect, bridging both micro and macrocracks. This strategy enabled the assessment of synergistic fibre interactions under identical matrix and admixture conditions. Detailed properties of all fibres used in the study are summarised in Table 2.

**Table 2:** Properties of fibres.

Fibre designation	PM38/PM12	PD38	B12	PVA12
Type	Polyolefin	Polypropylene	Basalt	Polyvinyl alcohol
Shape	Extruded	Crimped	Straight	Straight
Length, l (mm)	38/12	38	12	12
Diameter, d (mm)	0.45	0.30	0.013	0.010–0.025
Density (kg/m^3^)	910	910	2900	1300
Elastic modulus (GPa)	> 10	> 10	79–110	25–35
Tensile strength (MPa)	550–650	550–600	1200–1400	> 1650

The base mix was designed to ensure both high performance and self-compacting properties. It included a relatively high binder content, low water-to-binder ratio, and carefully balanced additives. All fibre-reinforced mixes were derived from this reference composition. The detailed proportions of all mixtures are presented in Table 3.

**Table 3:** Mix proportions (amounts per m^3^ of concrete).

Component	Symbol/unit	*R*	PM38	PD38	PM12	B12	PVA12	PD38 + PVA12	PD38 + B12	PM38 + B12
CEM I 52.5R cement	**C** (kg/m^3^)	750	750	750	750	750	750	750	750	750
Fly ash	**FA** (kg/m^3^)	100	100	100	100	100	100	100	100	100
Zeolite-based additive (Z-50)	**Z** (kg/m^3^)	100	100	100	100	100	100	100	100	100
Metakaolin (MK-40)	**M** (kg/m^3^)	50	50	50	50	50	50	50	50	50
Colloidal nanosilica	**SF** (L/m^3^)	50	50	50	50	50	50	50	50	50
Quartz sand (0.01–0.5 mm)	**S** (kg/m^3^)	1050	1050	1050	1050	1050	1050	1050	1050	1050
Admixtures	**As** (L/m^3^)	39	39	39	39	39	39	39	39	39
Water	**W** (L/m^3^)	310	310	310	310	310	310	310	310	310
Polyex Mesh fibre (l = 38 mm)	**PM38** (kg/m^3^)	—	13.65	—	—	—	—	—	—	6.83
	V_PM38_ (%)	—	1.5	—	—	—	—	—	—	0.75
Polyex Duro fibre (l = 38 mm)	**PD38** (kg/m^3^)	—	—	13.65	—	—	—	6.83	6.83	—
	V_PD_ (%)	—	—	1.5	—	—	—	0.75	0.75	—
Polyex Mesh fibre (l = 12 mm)	**PM12** (kg/m^3^)	—	—	—	13.65	—	—	—	—	—
	V_PM12_ (%)	—	—	—	1.5	—	—	—	—	—
Basalt fibre (l = 12 mm)	**B12** (kg/m^3^)	—	—	—	—	43.5	—	—	21.75	21.75
	V_B_ (%)	—	—	—	—	1.5	—	—	0.75	0.75
PVA fibre (l = 12 mm)	**PVA12** (kg/m^3^)	—	—	—	—	—	19.5	9.75	—	—
	V_PVA_ (%)	—	—	—	—	—	1.5	0.75	—	—

All mixtures were prepared in a 50 L pan mixer following a standardised sequence. Dry components were premixed for three minutes to ensure a uniform distribution. Subsequently, the mixture and water were gradually added, followed by further mixing. Fibres were slowly introduced to prevent clumping and ensure even distribution, with a final mixing time of five minutes. After homogenisation, the fresh concrete was immediately subjected to slump-flow, V-funnel, L-box and J-ring tests to evaluate workability, viscosity, passing ability, and potential for blockage. Each fresh-state test was performed once for each mixture, in accordance with standard SCC testing practice, and the reported values represent single measurements.

After completion of fresh property tests, the concrete was poured into moulds appropriate for further mechanical testing. Cubes with dimensions of 100 × 100 × 100 mm were prepared for compressive and splitting tensile strength tests, cylinders of 150 × 300 mm for determination of elastic modulus, and beams (80 × 140 × 740 mm) for evaluation of fracture performance. Immediately after casting, the upper surfaces of all samples were moistened and covered with plastic sheets to prevent excessive evaporation. After 48 h, the specimens were demoulded. For the next 7 days, they were kept under semi-sealed conditions, cyclically moistened and covered with foil to limit uncontrolled water loss. After this initial curing stage, all samples were stored under ambient laboratory conditions (approximately 20 ± 2 °C, relative humidity 50–65%) until the testing age of 28 days.

Prior to mechanical testing, specific surface treatments were applied to the samples to improve the precision of the testing. All cylinders were carefully ground using a surface grinder to obtain parallel ends and eliminate surface irregularities. In the case of beams, the notch for the fracture toughness testing was introduced using a precision diamond saw immediately before testing. The notch had a depth of 50 mm (approximately one-third of the beam height), a uniform width of approximately 3 mm, and was positioned centrally at mid-span. This approach ensured a clean and controlled notch geometry and minimised the possibility of clustering of fibres at the tip of the notch, which could otherwise interfere with crack initiation and propagation mechanisms.

### V-Funnel test for viscosity assessment

The viscosity of the HPSCC mixtures was evaluated using the V-funnel method according to EN 12350-9:2010^34^ and the EFNARC guidelines^35^. The test was performed to assess the flowability and resistance to segregation of the HPSCC mixes developed as part of this study. The apparatus consisted of a stainless steel V-shaped funnel with smooth inner surfaces and a watertight sliding gate at the base. Before each test, the funnel was thoroughly moistened with water to minimise surface friction. A fresh concrete sample was poured into the funnel without compaction, and the excess material was levelled with a straight edge to align with the upper rim.

After allowing the mixture to rest for 10 ± 2 s, the sliding gate was opened in one quick motion. The flow time of the V-funnel was measured from the moment the gate was opened until a vertical line of sight through the funnel became unobstructed. The discharged concrete was collected in a container with a minimum capacity of 12 L. The flow time was recorded with an accuracy of 0.1 s. Each mixture was tested within 10 min after mixing completion to ensure consistency of the result.

### L-Box test for passing ability of HPSCC

The L-box test is used to evaluate the passing ability of HPSCC, specifically its ability to flow through congested reinforcement without segregation or blocking. This method simulates conditions similar to those encountered in reinforced concrete elements. The procedure follows the guidelines outlined in PN-EN 12350-10:2011^36^ and the EFNARC recommendations^35^.

Before testing, all internal surfaces of the apparatus are moistened to reduce friction. The vertical section of the L-box is filled with freshly mixed HPSCC without any external compaction. After a rest period of 60 ± 10 s, the sliding gate separating the vertical and horizontal sections is raised, allowing the mixture to flow through the reinforcement bars into the horizontal channel.

The primary measured parameter is the passing ratio (PA), defined as the ratio of the average height of concrete at the end of the horizontal section (H₂) to the height of concrete behind the gate at the beginning of the test (H₁), as given by the Eq. 

1 $$PA=\frac{{{H_2}}}{{{H_1}}}$$

To ensure accuracy, both *H₁* and *H₂* are measured at three locations across the width of the channel (two outer points and one central point), and average values are used. The closer the *PA* ratio is to 1.0, the better the passing ability of the HPSCC mixture, indicating minimal blocking or segregation.

### J-ring test for passing ability and flow characteristics

The J-ring test is used to evaluate the ability of HPSCC to flow through reinforcement-like obstructions, while simultaneously assessing its flowability and segregation resistance. The method is based on the slump flow test but incorporates a ring of vertical steel bars placed around the cone to simulate reinforcement congestion.

As in the standard slump flow procedure, the cone is filled with fresh HPSCC without compaction. After a rest period, the cone is lifted vertically in a single motion, and the time required for the concrete to reach a diameter of 500 mm is measured as *T*_*500*_. The final flow spread is measured in two perpendicular directions and averaged as the J-Ring flow diameter (D_final_)^37^.

The presence of the ring allows visual observation of any signs of blockage, aggregate bridging, or fibre accumulation. The test provides an indication of both the flow rate and the passing ability.

**Fig. 2:**
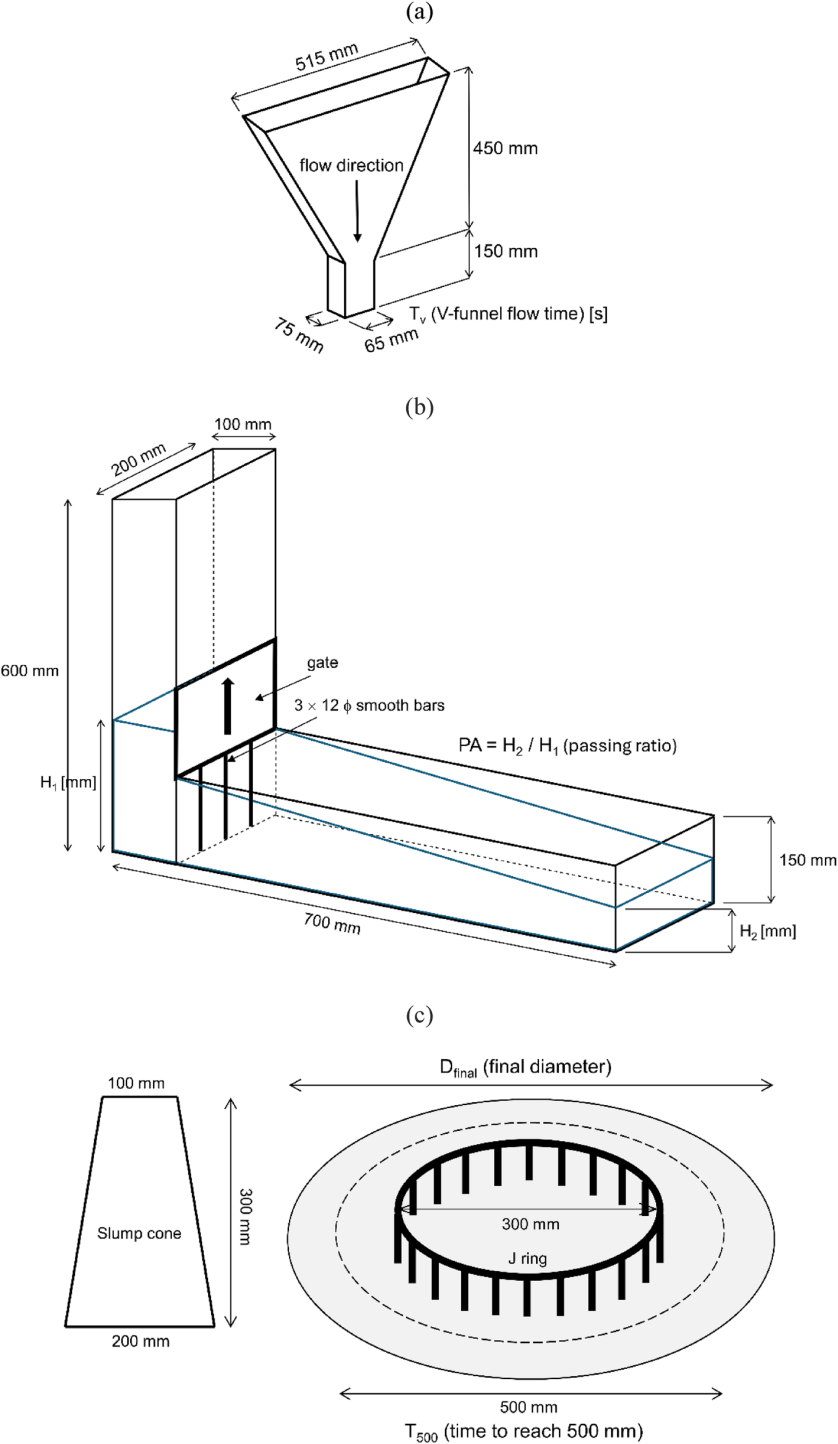
Simplified schematic diagrams of the fresh-state test setups used in this study according to EFNARC recommendations: (**a**) V-funnel test for determining the flow time (T_v_), (**b**) L-box test for evaluating passing ability expressed as blocking ratio (H_2_/H_1_), and (**c**) J-ring test for assessing passing ability based on the final slump-flow diameter (D_final_) and the time required to reach a diameter of 500 mm (T_500_).

For clarity, schematic diagrams of the V-funnel, L-box and J-ring test setups used in this study are provided in Fig. 2. The diagrams indicate the main geometrical features and measurement locations relevant for evaluating flowability, passing ability and blocking effects.

### Compressive strength

Compressive strength, defined as the maximum resistance of concrete to axial compressive load, was determined on cubic specimens measuring 100 × 100 × 100 mm. The tests were performed after 28 days of curing in accordance with PN-EN 12390-3:2019-07^38^. For each mixture, four samples were tested and the mean value was reported.

### Modulus of elasticity

The static modulus of elasticity, defined as the ratio of stress to corresponding strain within the elastic range, was measured according to PN-EN 12390-13:2021-12^39^, using cylindrical samples (150 mm in diameter × 300 mm in height) after 28 days of curing. The top and bottom surfaces of each specimen were ground flat to ensure uniform stress distribution. One specimen was tested for each concrete mix. Therefore, the reported modulus values represent single measurements.

### Splitting tensile strength

Splitting tensile strength was determined according to PN-EN 12390-6:2011^40^, using 100 × 100 × 100 mm cubic samples tested after 28 days. The load was applied along two opposite faces via 4 mm wide and 100 mm long fibreboard strips, centred along the specimen length. Five specimens per mix were tested.

### Flexural strength

**Fig. 3:**
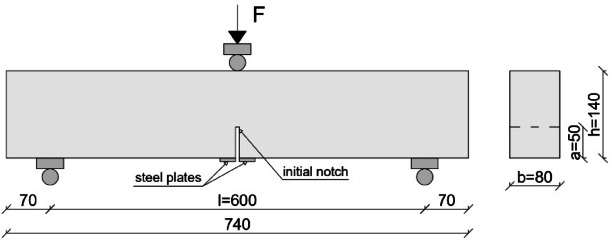
Test specimen (dimensions in mm).

Flexural strength tests were carried out on prismatic beams after 28 days of curing. Two specimens were tested for each mix, and the reported values represent mean results obtained from two specimens. The tests were carried out using a three-point bending configuration, with the load applied mid-span, according to PN-EN 12390-5:2019-08^41^. The dimensions of the samples and the loading scheme are shown in Fig. 3. To calculate the flexural tensile strength, the effective cross-sectional area was reduced by the length of the notch (50 mm) in the lower mid-span. This modified cross section was used to reflect the stress distribution near the notch under loading.

### Fracture parameters

Fracture behaviour was assessed on notched beam samples prepared as described in Fig. 3, using the same setup as in the flexural strength test. A central notch of 50 mm depth (approximately one-third of the beam height) was introduced after 21 days of curing. Steel plates were bonded symmetrically on both faces of the notch 48 h before testing to mount a clip gauge for measuring crack mouth opening displacement (CMOD).

The fracture energy (*G*_*f*_) was calculated based on the area under the load–deflection curve, following the recommendations of RILEM TC 50-FMC^42^ and RILEM TC 89-FMT^43^ for notched beams tested in bending. The effective fracture energy was determined using the Eq. 

2 $${G_f}=\frac{{{A_i}}}{{{b_i}{h_i}}}$$

where: *G*_*f*_ is the fracture energy normalised by the fractured ligament area [J/m^2^], *A*_*i*_​ is the area under the load–displacement curve [kN×mm], *b*_*i*_​ is the width of the specimen in the notch [mm], and *h*_*i*_is the effective height of the specimen in the notch [mm].

Fracture parameters were determined as mean values obtained from two notched beam specimens per mix. The fracture energy values were finally expressed in J/m^2^, following the RILEM recommendations, to ensure consistency with the reported results.

### Hybrid effect on fracture energy

The hybrid effect on the energy of the fracture was quantified using the hybrid index (α) proposed by Feng et al.^22^, defined as

3 $$\alpha =\frac{{{E_H} - {E_0}}}{{\sum {\left( {{E_i} - {E_0}} \right){\beta _i}} }}$$

where *β*_*i*_ *= V*_*i*_*/V* ​​is the volume fraction of type fibre *i* in the total fibre volume, *∑β*_*i*_ *=* 1, *E*_*H*_​ is the fracture energy of the hybrid fibre reinforced concrete, *E*_*0*_ is the fracture energy of plain concrete (no fibres), *E*_*i*​_ is the fracture energy of the mix of single-fibre concrete.

A value of α > 1 indicates a positive hybrid effect, while α < 1 suggests a negative or non-synergistic interaction, consistent with the criteria proposed by Song et al.^44^. It should be noted that the effect of fibre length is implicitly captured in the hybrid index through the fracture energy values *E*_*i*_, which were determined separately for each single-fibre system with a defined fibre type and length. Therefore, combinations of fibres with different lengths (e.g. 12 mm and 38 mm) inherently reflect length-dependent interactions via their individual fracture responses. The hybrid index does not introduce fibre length as an explicit variable, but evaluates whether the experimentally observed fracture energy of the hybrid system exceeds the weighted contribution of the corresponding single-length fibre systems.

Accordingly, the hybrid effect quantified in this study represents the combined interaction of fibre type and length under identical matrix conditions, rather than a purely volumetric superposition.

## Results and discussion

### Fresh-state properties

#### V-Funnel test for viscosity assessment

**Fig. 4:**
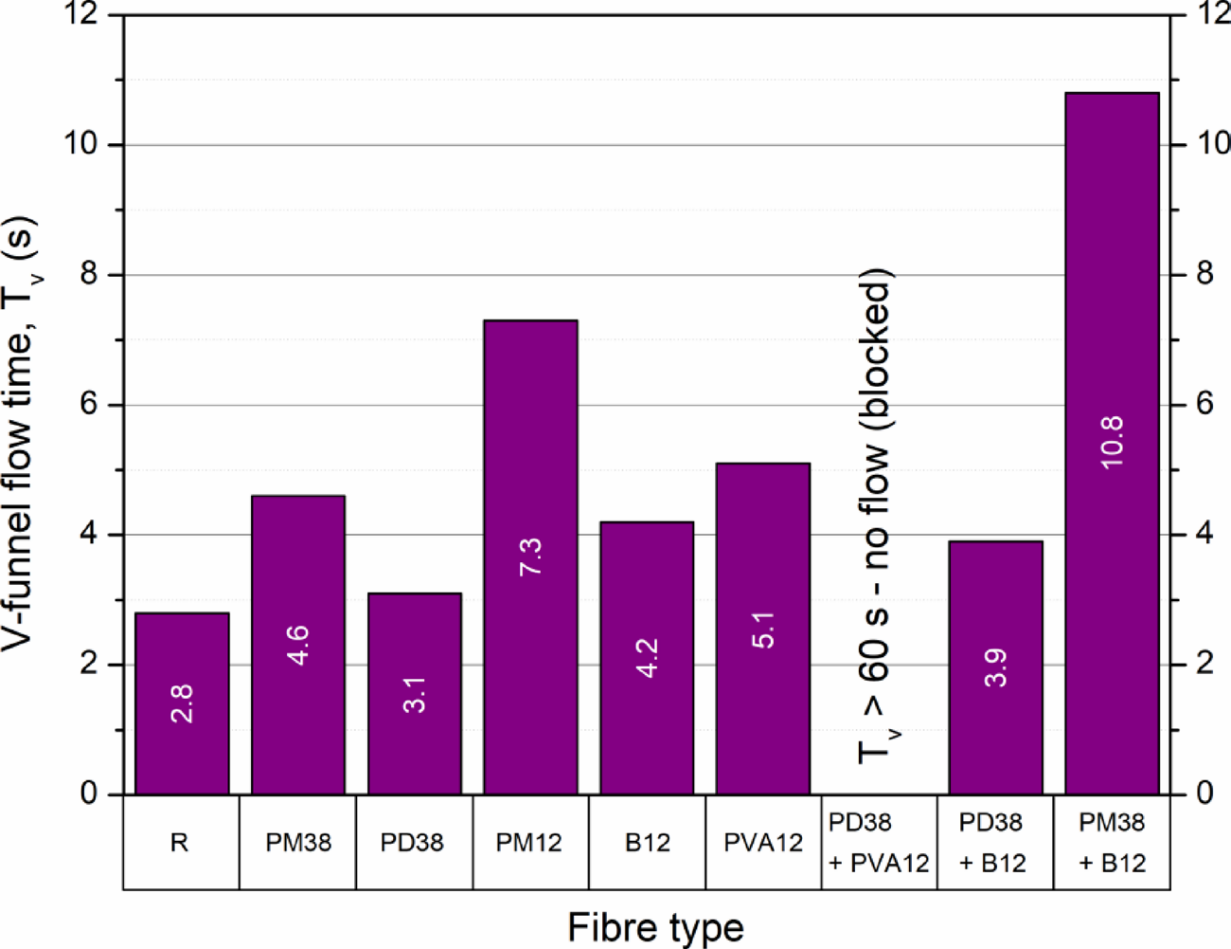
V-funnel flow time of concrete mixtures as a function of fibre type and combination. For PD38 + PVA12, no flow was observed due to blockage at the funnel outlet, therefore the V-funnel time could not be determined.

**Fig. 5:**
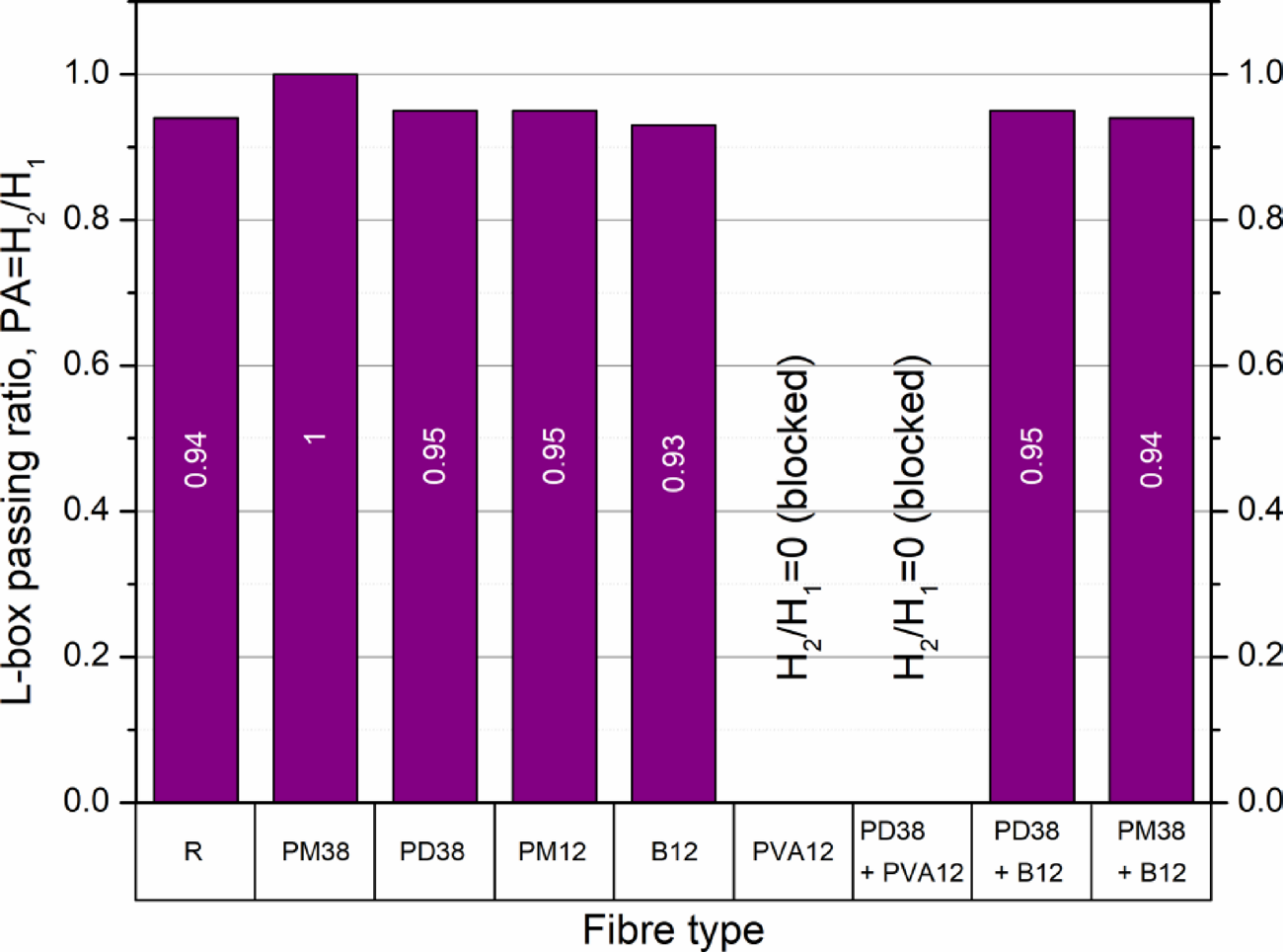
L-box passing ratio of concrete mixtures with different fibre types and combinations. For PVA12 and PD38 + PVA12, the mixture was blocked by the reinforcing bars and the test could not be completed; therefore. The blocking ratio is reported as H_2_/H_1_ = 0 (blocked).

**Fig. 6:**
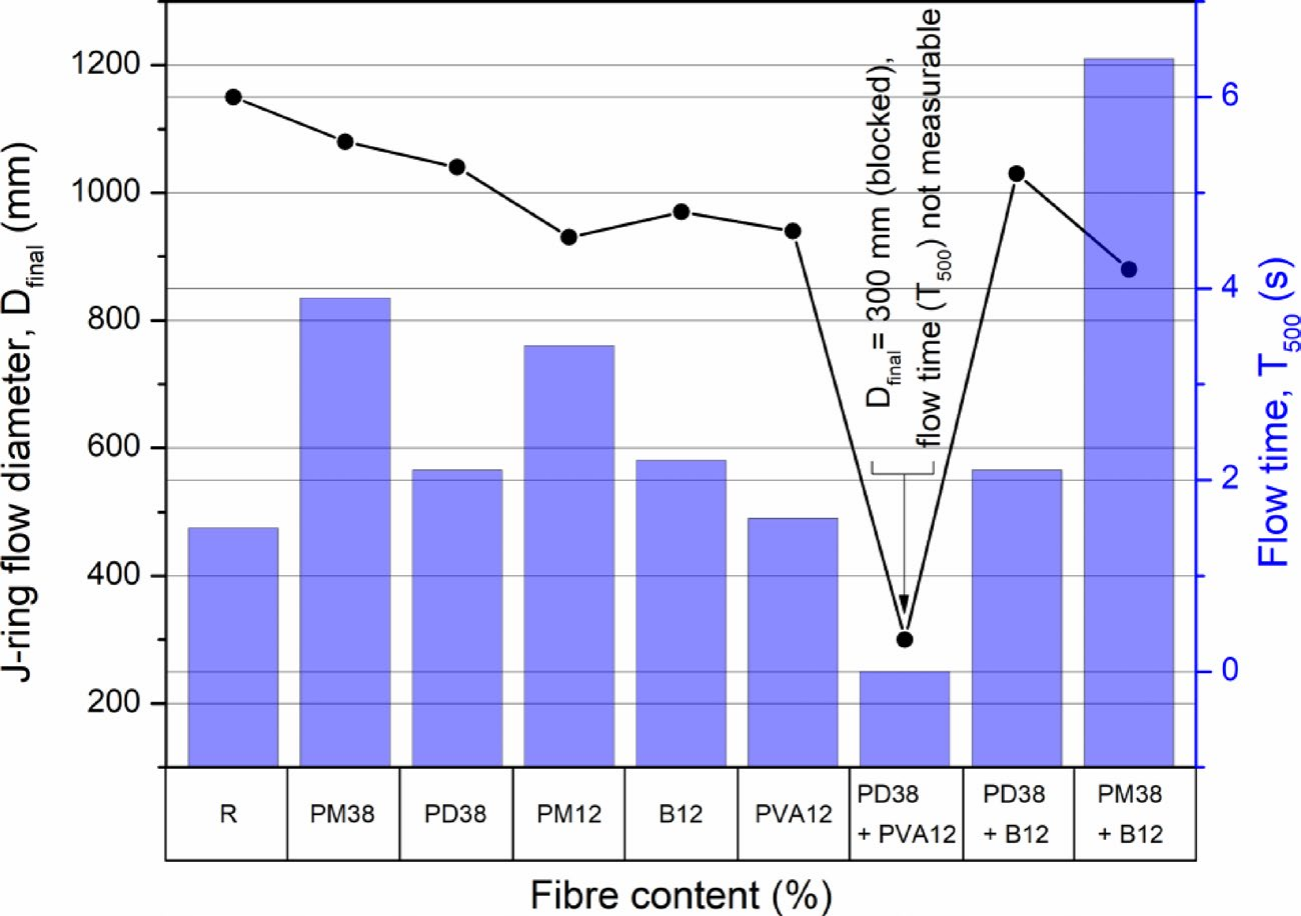
J-ring flow diameter and flow time of concrete mixtures with various fibre combinations. For PD38 + PVA12 slump-flow diameter below 500 mm (test could not be completed).

The results of the V-funnel test (Fig. 4) demonstrated that the incorporation of fibres significantly affects the viscosity of fresh concrete. For mixtures in which the V-funnel test and other fresh-state test could not be completed due to blockage, the corresponding results are reported using limiting values and explicit annotations in Figs. 4, 5 and 6. The control mix, free of any fibres, recorded the shortest flow time of 2.8 s, confirming its superior workability. The introduction of fibres generally resulted in reduced flowability and increased flow times, attributable to higher internal resistance and potential obstruction during discharge.

Based on fibre characteristics (Table 2), the influence of each type can be better understood. Polyolefin (PM) and polypropylene (PD) fibres have relatively low elastic moduli (> 10 GPa) and moderate tensile strengths (500–650 MPa), while basalt fibres (B12) are significantly stiffer (70–100 GPa) and stronger (1200–1400 MPa). PVA fibres, though short and thin, combine moderate stiffness (25–35 GPa) with low diameter, thus strongly interacting with the cement matrix.

Among the single-fibre mixes, PD38 (crimped polypropylene, 38 mm) showed a moderate increase in flow time to 4.6 s, suggesting an acceptable viscosity. On the contrary, PM12 (polyolefin, 12 mm) resulted in the highest increase among nonhybrid mixes (7.3 s, + 160.7%), likely due to its small size and high surface area, which improved the matrix-fibre interaction and resistance to flow. The PVA12 fibre mix showed a flow time of 5.1 s, which, although within the HPSCC limit (8–12 s), was higher than for most synthetic fibres. Given its extremely low diameter (0.010–0.025 mm) and moderate stiffness, the significant increase in viscosity is attributed to its high fibre count per unit mass and increased interface friction. Basalt fibres (B12) caused a moderate flow time of 4.2 s. Despite their high stiffness and strength, their smooth, straight geometry likely contributed to better dispersion and reduced mechanical interlock compared to synthetic crimped fibres.

Hybrid mixtures produced mixed outcomes. The mix of PD38 + B12 showed a relatively short flow time (3.9 s, + 39.3%), suggesting that the combination of a flexible crimped fibre (PD38) with rigid straight basalt fibres (B12) allowed optimal packing and reduced inter-fibre interference. This may have balanced structural reinforcement and workability. On the other hand, the PM38 + B12 hybrid exhibited the highest measurable flow time (10.8 s, + 285.7%), close to the HPSCC upper limit and risking segregation. This could be due to excessive fibre content and the combination of long fibres (38 mm) with high aspect ratio basalt fibres, leading to blockage in the V-funnel. The mix containing both PD38 and PVA12 did not discharge naturally, confirming excessive viscosity. The blend of crimped polypropylene and ultra-fine PVA12 fibres likely produced an entangled fibre network, dramatically increasing flow resistance. This outcome exceeds the recommended flow time for HPSCC, indicating the need for mix adjustments, such as an increased superplasticiser dosage or a water-to-binder ratio.

The results consistent with those of Çelik and Bingöl^25,45^, who observed that shorter fibres (12 mm) significantly increased flow times more than longer fibres (24 mm) at the same dosage. Polypropylene fibres, in particular, had a stronger impact than basalt, with increases of up to 84.7%. Similarly, Gencel et al.^46^ found that PP fibres above 0.33% compromised the SCC characteristics. Ghodousian et al.^47^ showed that even 0.1% PVA fibres could increase the flow time in a comparable way to 1.0% of 50 mm PP fibres, underlining the profound influence of fibre fineness. Diddi et al.^48^ demonstrated that 1% of 12 mm PP fibres caused a 58.3% increase in flow time, pushing the mix beyond the SCC limits.

In summary, the viscosity of fresh concrete is highly sensitive to the characteristics of the fibre. Shorter fibres with smaller diameters (especially PVA and polypropylene) have a disproportionate effect on increasing the flow time. Although stiff fibres like basalt moderately impact viscosity, their high density and straight geometry may facilitate better flow. The optimal hybrid composition, as seen in the PD38 + B12 mixture, can preserve workability while providing structural benefits. The type, shape, stiffness, and dosage of the fibre must be carefully balanced to retain the characteristics of HPSCC.

#### L-Box test for passing ability of HPSCC

The results of the L-box test (Fig. 5) reveal variations in the pass ability of concrete mixtures with fibre additions. The control sample recorded a ratio of 0.94, within the acceptable range for HPSCC. The highest value (1.00) was obtained with PM38 fibres, indicating no significant blockage and excellent flow through the apparatus.

Most fibre-reinforced mixtures maintained acceptable passing ratios between 0.93 and 0.95, suggesting that, when optimally dosed, fibre additions do not drastically alter the passing ability. However, the inclusion of PVA12 fibres, both alone (1.5%) and in hybrid form with PD38, caused the mixture to fall below the HPSCC requirement of 0.80, due to severe obstruction in the L-box. This pronounced reduction is attributed to the extremely small diameter (0.010–0.025 mm) and short length (12 mm) of PVA fibres, which significantly increase friction between particles and reduce flow through narrow gaps. Although the PD38 fibres alone (1.5%) slightly improved the ratio (0.95), the hybrid PD38 + PVA12 mixture completely failed to pass through the apparatus, indicating poor compatibility in terms of rheological behaviour.

These findings are consistent with previous studies. Çelik et al.^24,25^ reported that increasing the content of basalt and polypropylene fibres (12–24 mm) from 0.15% to 0.30% led to reductions in the L-box ratios from 0.94 to as low as 0.76. Similarly, Diddi et al.^48^ observed a drop from 0.94 to 0.86 with 1.0% polypropylene (12 mm). Longer fibres, such as those used in the studies by Guerini et al.^21^, caused smaller reductions, from 0.98 to 0.90, confirming that short and stiff fibres hinder flow more severely.

Aslani and Nejadi^49^ reported complete blockage in mixtures containing 0.55% 65 mm polypropylene fibres, although this could be due to inadequate initial workability. These outcomes underscore the importance of designing highly flowable base mixes when fibres are to be incorporated, especially those with small diameters or high stiffness.

In summary, while most fibre additions allow acceptable flow through the L-box, PVA fibres critically reduce passing ability and should be used with caution or in combination with appropriate rheology-enhancing admixtures.

#### J-Ring test for passing ability and flow characteristics

The J-ring test (Fig. 6) provided a comprehensive assessment of both the flowability and the passing ability of HPSCC mixtures modified with various fibres. The control mixture demonstrated the highest flow diameter (1150 mm) and the shortest flow time (1.5 s), confirming its excellent deformation capacity and unobstructed flow through the ring. The introduction of 38 mm polymer fibres (PM38 and PD38) moderately reduced the flow diameter (6.1–9.6%) and increased the flow time (2.1 to 3.9 s), but the mixtures still met the requirements of HPSCC, indicating a tolerable impact on rheology. In contrast, mixtures containing short synthetic fibres (12 mm), particularly polyolefin and polyvinyl alcohol (PVA), exhibited a more pronounced deterioration of flow characteristics. These fibres led to increased resistance to flow due to the formation of dense fibre networks, reflected in lower spread diameters and longer flow times.

Among hybrid systems, the combination of crimped polypropylene (PD38) and basalt (B12) fibres yielded the most favourable result, with a flow diameter of 1030 mm and a flow time of 2.1 s. This suggests an effective synergy between the flexible geometry of synthetic fibres and the high stiffness of mineral fibres, resulting in improved internal lubrication and reduced mechanical interlock. On the contrary, the mixture incorporating PM38 and B12 achieved the lowest flow diameter (880 mm) and the highest flow time (6.4 s), indicating significant blockage. The most critical performance drop was observed in the PD38 + PVA12 system, which failed to meet the minimum required spread, likely due to severe fibre entanglement and interaction with the steel ring bars.

The observed trends are consistent with the previous literature. Çelik and Bingöl^45^ reported that both basalt and polypropylene fibres reduced the spread of the J-ring by up to 12.5% and 20.8%, respectively. Ponikiewski and Katzer^50^ found that even a small dosage (0.2%) of PVA12 fibres could lead to a dramatic loss in flowability (35% spread reduction), while longer basalt and polypropylene fibres caused more moderate but still significant effects. Gencel et al.^46^ and Aslani and Nejadi^49^ further confirmed that increased fibre dosage leads to higher flow resistance and longer flow times, although longer and more flexible fibres were generally less detrimental.

These findings underscore the importance of fibre selection and configuration in HPSCC design. While long polymer fibres can preserve flow characteristics when properly dosed, short or high-aspect-ratio fibres, especially when used in hybrid systems, may substantially limit the deformability and passing ability of the mix. Particular attention must be paid to the use of fine-diameter fibres such as PVA, which, despite their low content, exhibit a disproportionate impact on rheology and flow obstruction due to fibre dispersion and interlocking mechanisms.

For clarity, a summary of compliance with EFNARC/EN criteria for fresh-state tests (V-funnel, L-box and J-ring) is provided in Table 4. The table indicates whether each mixture meets the relevant passing ability and flow requirements based on the results presented in Sect. 3.1.1–3.1.3.

**Table 4:** Compliance of HPSCC mixtures with EFNARC/EN criteria for fresh-state tests.

Test	*R*	PM38	PD38	PM12	B12	PVA12	PD38 + PVA12	PD38 + B12	PM38 + B12
V-funnel	+	+	+	+	+	+	−	+	+
L-box	+	+	+	+	+	−	−	+	+
J-ring	+	+	+	+	+	+	−	+	+

Note: ”+” meets EFNARC/EN criteria; ”–” does not meet criteria.

### Hardened properties

#### Compressive strength

**Fig. 7:**
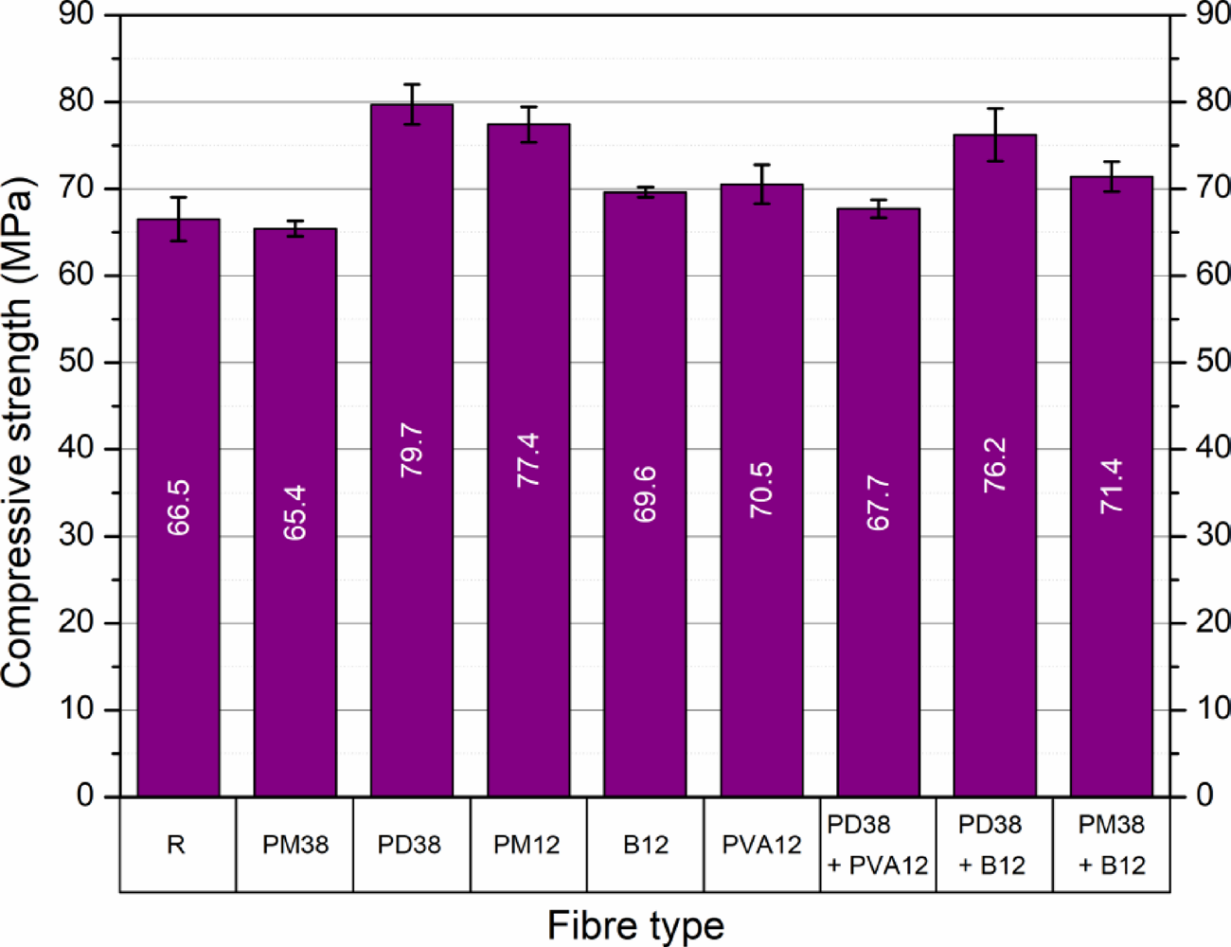
Compressive strength of HPSCC with different fibre types and combinations.

The compressive strength results of HPSCC incorporating different single and hybrid fibre systems are summarised in Fig. 7; Table 5. The control mix (R) achieved an average compressive strength of 66.5 MPa. All fibre-reinforced mixes, except one, showed enhanced strength, with improvements ranging from 1.8% to 19.9%, depending on fibre type, geometry and hybrid configuration.

**Table 5:** Statistical dispersion and relative changes in compressive strength of HPSCC reinforced with different fibre types and combinations.

Fibre type	SD (MPa)	CV (%)	Increase/decrease (%)
R	2.90	4.36	0.00
PM38	0.93	1.42	−1.65
PD38	2.71	3.40	+ 19.85
PM12	2.28	2.95	+ 16.39
B12	0.69	1.00	+ 4.66
PVA12	2.43	3.45	+ 6.02
PD38 + PVA12	1.02	1.51	+ 1.80
PD38 + B12	3.58	4.70	+ 14.59
PM38 + B12	2.02	2.83	+ 7.37

The greatest strength enhancement was observed for the mix containing PD38 fibres, a 38 mm crimped polypropylene fibre with a tensile strength of 500–580 MPa and a modulus greater than 10 GPa. The compressive strength reached 79.7 MPa, representing a 19.9% improvement. The crimped geometry and moderate stiffness enabled efficient crack bridging and fibre–matrix interaction, promoting energy absorption and delaying crack propagation. These results are consistent with the findings of Abousnina et al.^51^, where properly dispersed synthetic fibres improved concrete strength due to homogeneous distribution and improved post-cracking behaviour. The mix with PM12 fibres, a 12 mm extruded polyolefin fibre, achieved 77.4 MPa (+ 16.4%). Its shorter length and relatively low stiffness (modulus > 10 GPa) favoured uniform dispersion, minimising the formation of voids or clustering of fibres. On the contrary, PM38 fibres of the same material but longer (38 mm) slightly reduced the strength to 65.4 MPa, indicating that longer flexible fibres can entangle or disrupt the homogeneity of the mix if not well integrated.

Among mineral and synthetic straight fibres, basalt fibres (B12) and polyvinyl alcohol fibres (PVA12) improved their strength to 69.6 MPa and 70.5 MPa, respectively. Basalt fibres (12 mm, ρ = 2700 kg/m³) offer very high tensile strength (1200–1400 MPa) and modulus (70–100 GPa), allowing efficient microcrack control and better load redistribution. PVA fibres (12 mm, tensile strength 170–260 MPa, modulus 25–35 GPa), although less stiff, are known for excellent bonding to the cementitious matrix and contributed to the densification of the interfacial transition zone.

In hybrid systems, the best performing mix was PD38 + B12, which reached 76.2 MPa (+ 14.6%). This synergy probably resulted from combining long ductile macrofibres with short, stiff microfibres, where PD38 fibres contributed to post-cracking ductility and B12 fibres provided bridging and confinement at the microstructural level. Çelik and Bingöl^45^ and Deng et al.^52^, who highlighted the complementary roles of synthetic and mineral fibres, noted a similar hybrid advantage. Interestingly, the PD38 + PVA12 mix produced the lowest hybrid performance (67.7 MPa), nearly equal to the control. Despite the high fibre-matrix adhesion of PVA12 fibres and the effectiveness of PD38 fibres on their own, this combination may have been suboptimal due to conflicting deformability and insufficient synergetic action, or due to loss of workability and air entrainment that affects the uniformity of the mix - issues reported in similar fibre combinations by Fu et al.^53^.

All mixes demonstrated acceptable repeatability, with standard deviations ranging from 0.7 to 3.6 MPa and coefficients of variation generally below 4%, confirming the consistency of the results. The control mix exhibited the highest variability, which may reflect the absence of fibres to constrain the effects of internal cracking and shrinkage.

Current findings are consistent with the literature reporting initial strength gains with fibre additions, followed by plateaus or reductions at higher volumes or when dispersion is suboptimal. The observed performance hierarchy - PD38 > PM12 > PD38 + B12 > PM38 + B12 > PVA12 > B12 > PD38 + PVA12 > PM38 - highlights the importance of not only the type of fibre, but also its geometry, mechanical compatibility with the cement matrix, and the ability to maintain uniform distribution. It should be noted that basalt fibres, despite their excellent mechanical properties, contributed only moderately to compressive strength. This supports the view that compressive strength is more influenced by fibre dispersion and bridging at the beginning of microcracks, rather than by tensile capacity alone.

A one-way analysis of variance (ANOVA) was performed to examine whether the type of fibre reinforcement significantly affected the compressive strength of high-performance self-compacting concrete (HPSCC). The analysis revealed a statistically significant effect of fibre type on compressive strength, with an F-value of 19.65 and a corresponding p-value less than 0.0001 (Table 6). This outcome confirms that the mean compressive strength values of at least some fibre-reinforced mixes differ significantly from others within the 95% confidence level.

**Table 6:** Summary of one-way ANOVA for compressive strength of concrete with different fibre types.

Source	Sum of squares	Degrees of freedom	Mean square	F	*p*-value
Fibre type	640.88	8	80.11	19.65	< 0.0001
Residual	109.59	27	4.06		
Total	750.47	35			

To further determine which specific mixes differ from each other, the Tukey Honestly Significant Difference (HSD) test was used for post hoc comparisons. The results showed that concrete reinforced with crimped polypropylene fibres (PD38) exhibited significantly higher compressive strength than most other groups, including PM38, PVA12 and the hybrid combination of PD38 + PVA12. Similarly, the mix containing short polyolefin fibres (PM12) differed significantly from PM38 and PVA12, highlighting the favourable impact of shorter fibres on the development of strength. On the contrary, concrete with long polyolefin fibres (PM38) showed significantly lower strength than PD38 and PM12, which is attributed to the potential difficulties in dispersion and orientation of longer flexible fibres within the matrix. No statistically significant differences were found between mixes containing basalt fibres (B12), polyvinyl alcohol fibres (PVA12) and the hybrid system PD38 + PVA12, suggesting comparable performance levels among these groups. The statistically significant contrasts between fibre groups were identified based on the confidence intervals obtained from the Tukey HSD test.

These findings confirm that the geometry, type and mechanical properties of the fibres (as described in Table 2) all influence the compressive performance. Crimped polypropylene fibres (PD38) and short polyolefin fibres (PM12) provided the most substantial and statistically reliable improvements.

**Fig. 8:**
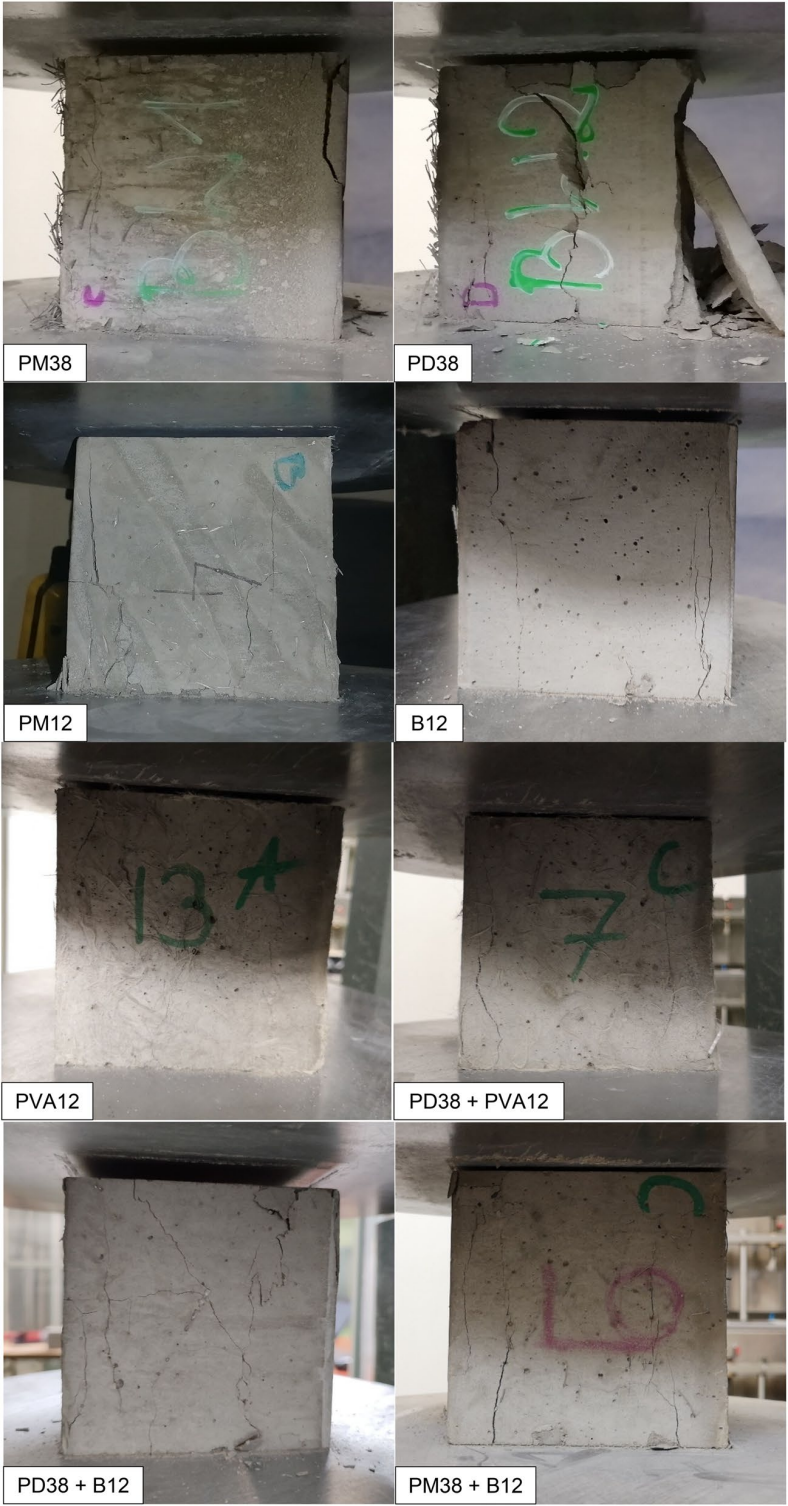
Failure modes of HPSCC samples reinforced with various types of fibre under uniaxial compression. Sample series are identified in the lower left corner of each image.

In addition to the statistical and mechanical evaluation, a qualitative visual analysis of the failure surfaces was conducted to evaluate the cracking behaviour and failure mechanisms of the HPSCC reinforced with different fibres. Each specimen was documented immediately after uniaxial compression and the fracture morphology was compared across all series (Fig. 8).

The specimen reinforced with PM38 fibres (polyolefin, 38 mm, extruded) exhibited a semi-brittle failure mode characterised by a dominant vertical splitting. The cracks were relatively wide and mostly unbranched, extending linearly from top to bottom, with only limited secondary cracking observed. This fracture pattern indicates a reduced contribution of fibres to crack deflection and post-cracking energy dissipation. At the macroscopic scale, the fracture surface appeared relatively clean, with only limited fibre-related features visible. While the fibre length and flexibility may influence fibre distribution and effectiveness, definitive conclusions regarding fibre dispersion or entanglement cannot be drawn based solely on macroscopic observations. The observed failure behaviour is consistent with the lower compressive strength and overall weaker mechanical performance of this series.

On the contrary, the sample containing PD38 fibres (crimped polypropylene, 38 mm) demonstrated a highly ductile failure mode. The fracture surface was rough and irregular, with visible oblique and transverse crack paths. At the macroscopic scale, fibre-related features were observed along the main crack trajectory, indicating the involvement of fibres during crack propagation. The crimped geometry of the PD38 fibres is expected to enhance frictional resistance and promote crack deflection and branching. However, detailed fibre–matrix interaction mechanisms cannot be conclusively identified without microscopic analysis. This macroscopic behaviour is consistent with the highest average compressive strength among all series and supports the effectiveness of crimped fibres in enhancing post-peak ductility and energy dissipation.

The specimen with PM12 fibres (polyolefin, 12 mm) showed a moderate ductile response. Several cracks propagated diagonally and in parallel, with occasional branching observed. The fracture surface exhibited a textured pattern, indicating increased surface roughness and the presence of multiple fracture planes at the macroscopic scale. The shorter fibre length may have facilitated more effective fibre distribution and interaction with the matrix; however, detailed fibre–matrix interaction mechanisms cannot be conclusively identified without microscopic analysis. The observed failure behaviour is consistent with the statistically significant improvement in compressive strength and suggests a balanced contribution to post-cracking response.

The failure pattern in the B12-reinforced specimen (basalt, 12 mm) was more restricted. Cracks were relatively narrow and distributed, but predominantly followed vertical paths. The fracture surface appeared comparatively smooth, with limited crack branching and no clearly distinguishable fibre pull-out features at the macroscopic scale. This behaviour may be associated with fibre rupture or interfacial debonding occurring at relatively low strains. However, such mechanisms cannot be directly confirmed without microstructural investigation. The stiff and brittle nature of basalt fibres, together with their small diameter, is consistent with a more localised energy dissipation process.

Concrete with PVA12 fibres (polyvinyl alcohol, 12 mm) exhibited a fracture behaviour similar to that observed for the B12 mixture. Fine, relatively evenly spaced cracks were observed, and the fracture surface appeared homogeneous at the specimen scale. PVA fibres are known to exhibit strong chemical affinity with cementitious matrices. Nevertheless, the absence of visible fibre pull-out in macroscopic images does not allow definitive conclusions regarding fibre rupture or interfacial failure. The observed crack pattern suggests a relatively uniform stress redistribution and limited crack opening.

In the PD38 + PVA12 hybrid system, a less favourable fracture mode was observed. A singular vertical crack dominated the failure surface, with minimal branching or lateral cracking. This behaviour reflects limited crack deflection and redistribution during bending. The fracture surface was characterised by a dominant crack path and an abrupt loss of load-carrying capacity. The statistical data also confirmed that this hybrid combination did not offer an improvement over the control group.

On the other hand, the PD38 + B12 hybrid revealed a highly desirable failure mode. Multiple cracks branched diagonally and vertically, with visible signs of fibre engagement. The rough surface of the fracture, irregular crack paths and fibre-related features indicate an effective interaction between the stiff basalt fibres and the ductile PD38 macrofibres at the specimen scale. This mixture demonstrated enhanced energy dissipation, which is consistent with its high compressive strength and favourable post-peak performance.

Finally, the PM38 + B12 mix showed intermediate behaviour. The fracture surface exhibited increased roughness compared to PM38 alone, with more pronounced crack deviation and occasional branching. Fibre pull-out features were visible, particularly in regions associated with basalt fibres. This observations indicate that the addition of B12 modified the fracture behaviour of PM38-reinforced concrete, although the extent of this modification was lower than that observed in the PD38 + B12 system.

Overall, visual observations show that fracture morphology in fibre-reinforced concrete is strongly influenced by fibre geometry, length, and fibre–matrix interaction. Ductile failure modes, characterised by multiple crack paths, crack deflection, and visible fibre engagement, were observed in the PD38, PM12 and PD38 + B12 mixes. Brittle or semi-brittle patterns, with dominant single cracking and limited crack branching, were found in PM38, PVA12 and PD38 + PVA12 mixes. These observations are consistent with the statistical trends in compressive strength and highlight the importance of optimising the type of fibre and hybridisation to tailor fracture behaviour in high-performance concrete.

#### Modulus of elasticity

**Fig. 9:**
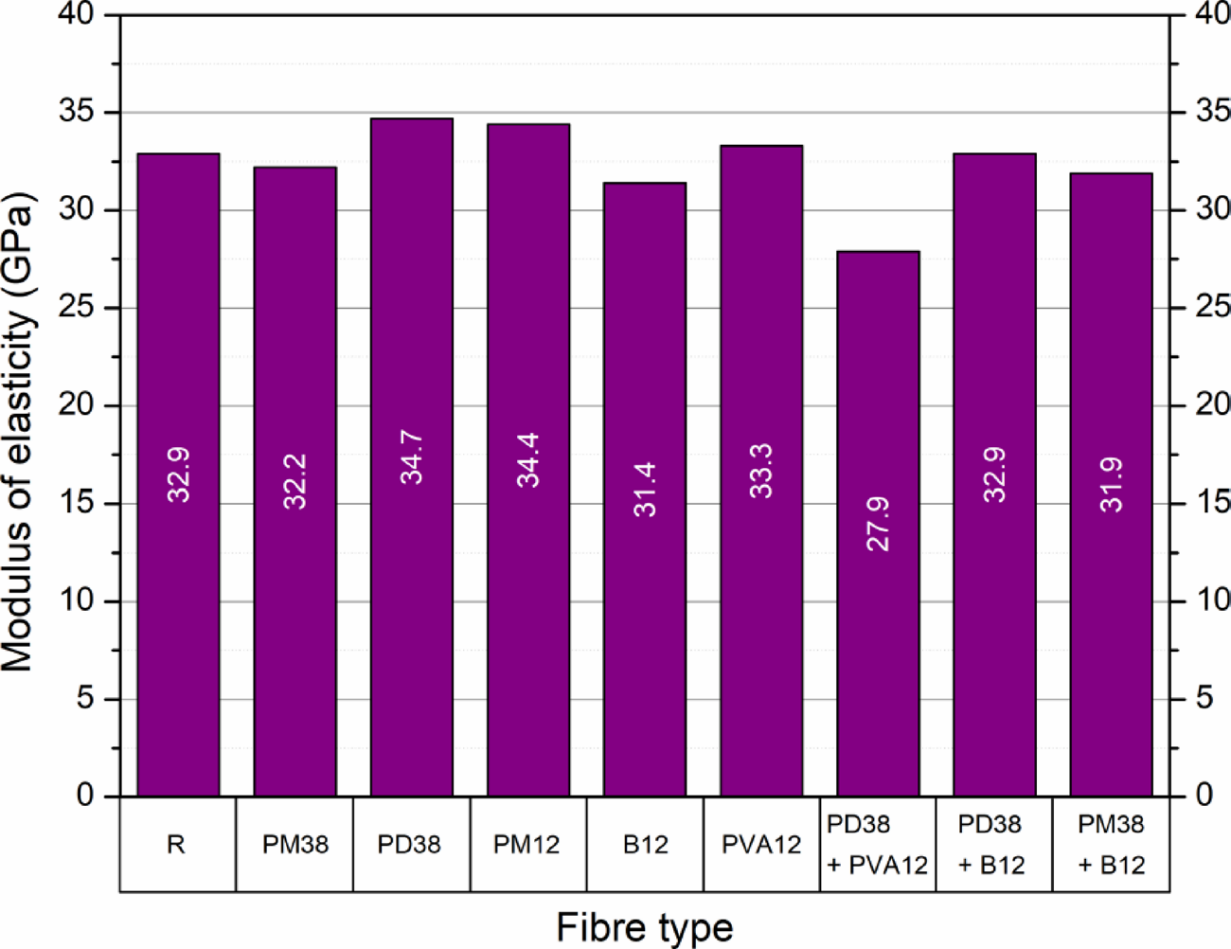
Modulus of elasticity of HPSCC with different types of fibres and combinations.

The results of the modulus of elasticity tests for the nine HPSCC mixes are presented in Fig. 9. The reference concrete (R) achieved a modulus of 32.9 GPa, which served as the baseline to assess the influence of fibre additions. Among the reinforced mixtures, both increases and reductions in modulus were observed, depending on the type and characteristics of the fibres used.

The highest modulus was recorded for the mix incorporating PD38 fibres (crimped polypropylene, 38 mm), reaching 34.7 GPa, which corresponds to a 5.5% increase compared to the control. A similar increase was observed for the PM12 fibre-reinforced mix (polyolefin, 12 mm), which achieved a modulus of 34.4 GPa (+ 4.6%). In both cases, the increase in compressive strength was accompanied by an increase in elastic stiffness, in line with the commonly reported correlation between these two parameters. The addition of PVA fibres (12 mm, straight) also resulted in a moderate increase in the modulus to 33.3 GPa (+ 1.2%). On the contrary, a decrease in modulus was observed for four of the mixes. The use of PM38 fibres (polyolefin, 38 mm, extruded) resulted in a value of 32.2 GPa (− 2.1%). The B12 fibre mix (basalt, 12 mm) reached 31.4 GPa (− 4.6%).

A marked reduction in modulus was found in the hybrid mix PD38 + PVA12, which decreased to 27.9 GPa, indicating a 15.2% decrease. The PM38 + B12 mix also showed a lower modulus of 31.9 GPa (− 3.0%). In contrast, the PD38 + B12 hybrid yielded a modulus of 32.9 GPa, identical to the reference.

These findings are in agreement with trends reported in previous studies. Gencel et al.^46^ observed increases in modulus of up to 5.5% in polypropylene fibre reinforced concretes, while Ayub et al.^54^ reported that basalt fibres generally led to limited changes in elastic stiffness. Similarly, Aslani and Nejadi^49^ showed that variations in modulus associated with polypropylene fibres may depend on fibre type and curing age.

Overall, the results indicate that the modulus of elasticity of fibre-reinforced HPSCC varies with fibre type and mixture composition. Mixes that exhibited higher compressive strength generally showed higher elastic stiffness, supporting the empirical relationship between these two parameters reported in the literature^51^.

#### Splitting tensile strength

**Fig. 10:**
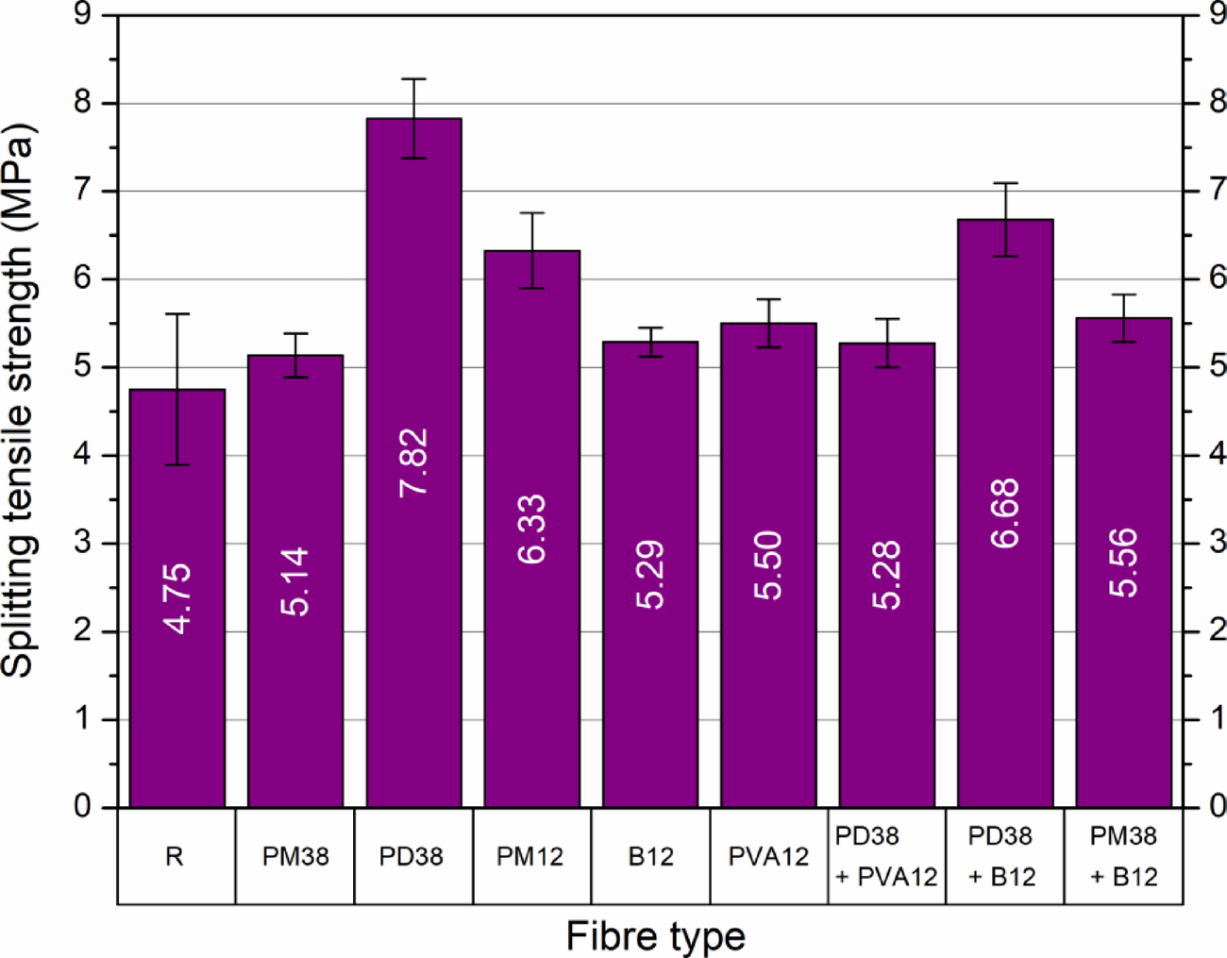
Splitting tensile strength of HPSCC with different fibre types.

The average results of the splitting tensile strength tests for nine HPSCC mixes are shown in Fig. 10. Each data point represents the mean value derived from five independent samples. The control mix (R) achieved a splitting tensile strength of 4.75 MPa, while all fibre-reinforced mixes exhibited improvements ranging from 8.2% to 64.8% relative to the control.

Among the mixes containing only a single fibre type, the greatest increase was observed for the PD38 mix (crimped polypropylene, 38 mm), which reached 7.82 MPa, corresponding to a 64.8% increase. This notable improvement is associated with the crimped geometry of the fibre, which enhances mechanical anchorage and crack-bridging capability during tensile loading. The mixture containing PM12 fibres (polyolefin, 12 mm, extruded) also achieved a substantial improvement (6.33 MPa, 33.3%), outperforming its counterpart with longer fibres (PM38, 5.14 MPa, 8.2%). This result highlights the favourable performance of short fibres in fibre-reinforced cementitious composites. Their reduced length may facilitate a more homogeneous distribution within the matrix, thereby limiting the formation of localised weak zones under tensile stress.

In terms of hybrid compositions, the combination of PD38 and B12 fibres resulted in a strength of 6.68 MPa, marking a 40.6% increase over the control. This hybrid system combines the crack-bridging capacity of macro-synthetic fibres (PD38) with the tensile contribution of basalt microfibres (B12). The combined effect resulted in enhanced tensile resistance compared to the individual fibre systems. Interestingly, the hybrid mix PM38 + B12 also showed improved tensile strength (5.56 MPa, 17.1%), despite the relatively poor performance of PM38 alone. This observation indicates that the inclusion of B12 fibres modified the tensile response of the PM38-reinforced concrete.

The remaining fibre-reinforced mixes (B12, PVA12, and PD38 + PVA12) all showed moderate improvements in tensile performance, ranging between 11.3% and 15.8%. Despite differences in fibre type and geometry, these mixes exhibited comparable levels of tensile enhancement relative to the control.

A clear correlation was observed between the increase in splitting tensile strength and the compressive strength performance of the same mixes. In general, fibre combinations that significantly improved compressive strength also produced higher tensile resistance. This reinforces the conclusion that fibre reinforcement improves the overall mechanical integrity of HPSCC, provided that the fibre type and mixture composition are appropriately selected.

These results are consistent with prior research. Gencel et al.^46^ reported a 5.1–49.4% increase in splitting tensile strength for polypropylene fibres (45 mm) at dosages between 0.33% and 1.32%, attributing the improvement to good fibre dispersion and pull-out resistance. Algin and Ozen^11^ and Ayub et al.^54^ both highlighted the role of basalt fibres, with optimal tensile strength observed for fibres of 24–25 mm in length and fibre contents up to 3%. Their findings suggest that longer basalt fibres offer better bridging performance and pull-out resistance compared to shorter variants. Çelik and Bingöl^25^ demonstrated splitting strength gains of 7.1–16.8% with basalt fibres (24 mm) and 5.8–14.2% with polypropylene fibres (19 mm). Smarzewski^5^ observed even greater increases (50.1–70.2%) with polypropylene fibres (12 mm) at 1–2% content, which was attributed to the high fibre count per unit volume associated with low-density fibres. The authors also reported diminishing returns with increasing basalt fibre content, which aligns with the findings of this work where hybrid mixes with excessive stiffness or poor dispersion showed reduced efficacy.

In summary, the splitting tensile strength of HPSCC is significantly influenced by the type, length, geometry, and hybridisation strategy of the fibre. Crimped polypropylene fibres (PD38) and short polyolefin fibres (PM12) proved to be the most effective in improving tensile capacity, while hybrid systems such as PD38 + B12 offered promising synergetic performance. The overall data confirm that proper selection and proportioning of fibres can significantly enhance the tensile response of high-performance self-compacting concretes.

The statistical analysis confirmed that the addition and type of fibres had a significant effect on the tensile strength of HPSCC. A one-way ANOVA test indicated a highly significant difference between the groups (F = 22.20, *p* < 0.001), validating that the observed differences were not due to random variation. The detailed results are summarised in Table 7.

**Table 7:** One-way ANOVA results for splitting tensile strength.

Source	Sum of squares	Degrees of freedom	F value	*p*-value
C(Fibre_type)	30.4130	8.0000	22.2047	0.0000
Residual	5.1362	30.0000	NaN	NaN

**Fig. 11:**
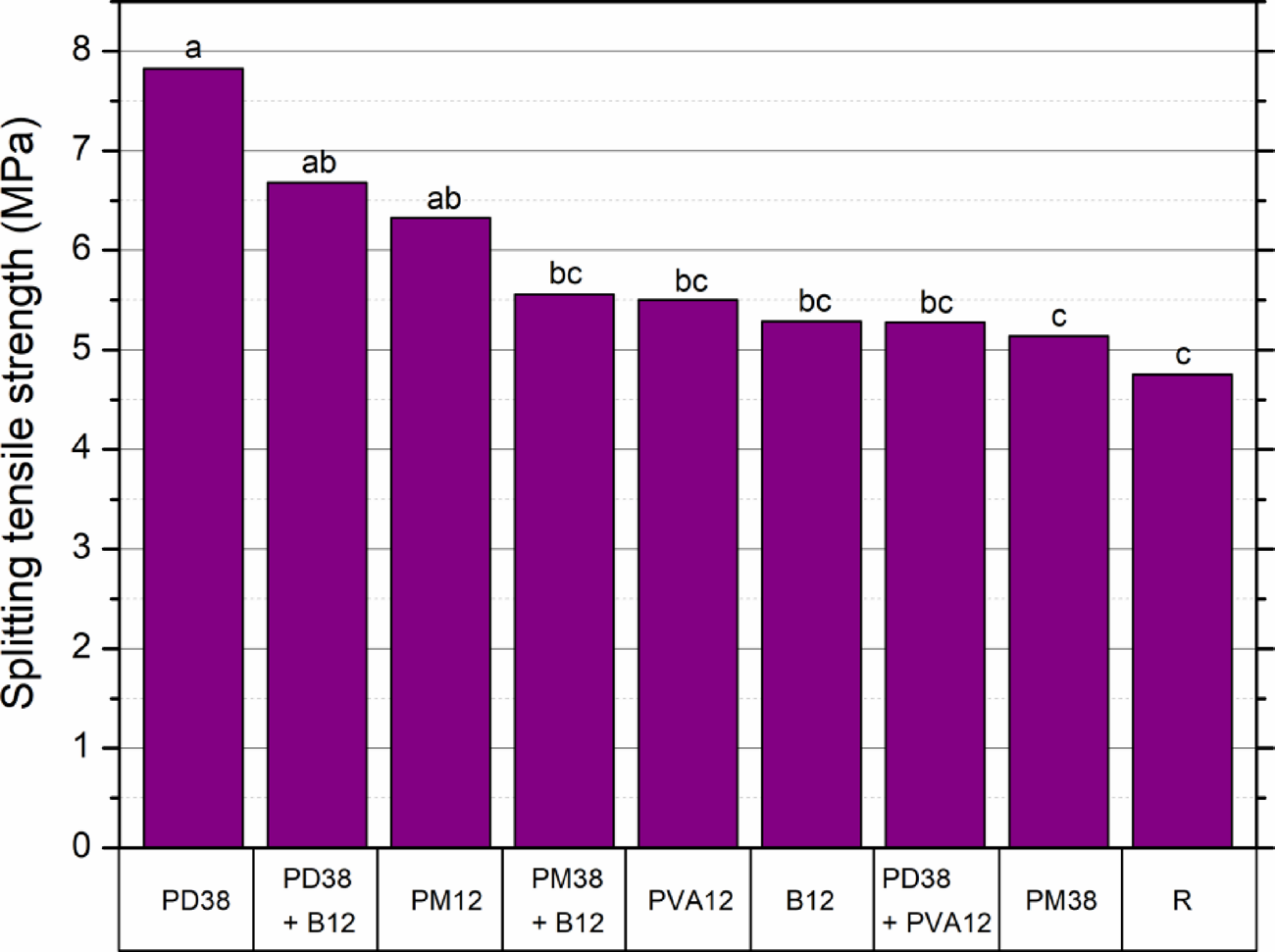
Mean splitting tensile strength of HPSCC with different types and combinations of fibres. The letters above the bars indicate statistically homogeneous groups according to Tukey’s HSD test (α = 0.05).

To identify which mixes differed significantly, Tukey’s post-hoc HSD test was conducted (Fig. 11). The analysis revealed that the PD38 mixture formed an isolated group (a), statistically distinct from all other series, confirming its dominant influence on tensile performance. The PD38 + B12 and PM12 mixes were grouped as ab, indicating that their improvements, although substantial, were not significantly different from each other, but still superior to the control.

Mixes such as PM38 + B12, PVA12, B12, and PD38 + PVA12 were grouped under bc, suggesting moderate enhancements that were not statistically distinct. Meanwhile, PM38 and the control group R belonged to the lowest tier (c), confirming their significantly lower performance compared to the PD38 dominant systems.

The descriptive statistics in Table 8 also reinforce these findings. The highest average splitting strength (7.82 MPa) and the highest relative improvement (64.7%) were achieved by the PD38 series. The lowest coefficient of variation (CV = 5.2%) was also recorded for this group, indicating excellent consistency between test specimens. In contrast, the control mix showed both the lowest mean strength (4.75 MPa) and a relatively high variability (CV = 18.1%).

**Table 8:** Statistical dispersion and relative changes in splitting tensile strength.

Fibre type	SD (MPa)	CV (%)	Increase (%)
R	0.86	18.07	0.00
PM38	0.25	4.86	8.16
PD38	0.45	5.75	64.74
PM12	0.43	6.75	33.16
B12	0.17	3.12	11.32
PVA12	0.27	4.94	15.79
PD38 + PVA12	0.28	5.22	11.05
PD38 + B12	0.41	6.21	40.63
PM38 + B12	0.27	4.86	17.05

In particular, the PM12 series achieved a strong balance between performance and reliability, with an average of 6.33 MPa (33.2%) and a low CV of 6.75%. In contrast, mixes with hybrid fibre systems exhibited moderate gains; the mix PD38 + B12 reached 6.68 MPa (40.6%) but showed greater variability (CV = 6.2%).

In general, these results clearly demonstrate the positive effect of the addition of fibres, particularly PD38 and its combinations, on the enhancement of the tensile behaviour of HPSCC. The alignment between statistical significance and mechanical performance strengthens the conclusion that fibre type and geometry critically govern the improvement in tensile strength in high-performance concrete.

#### Flexural strength

**Fig. 12:**
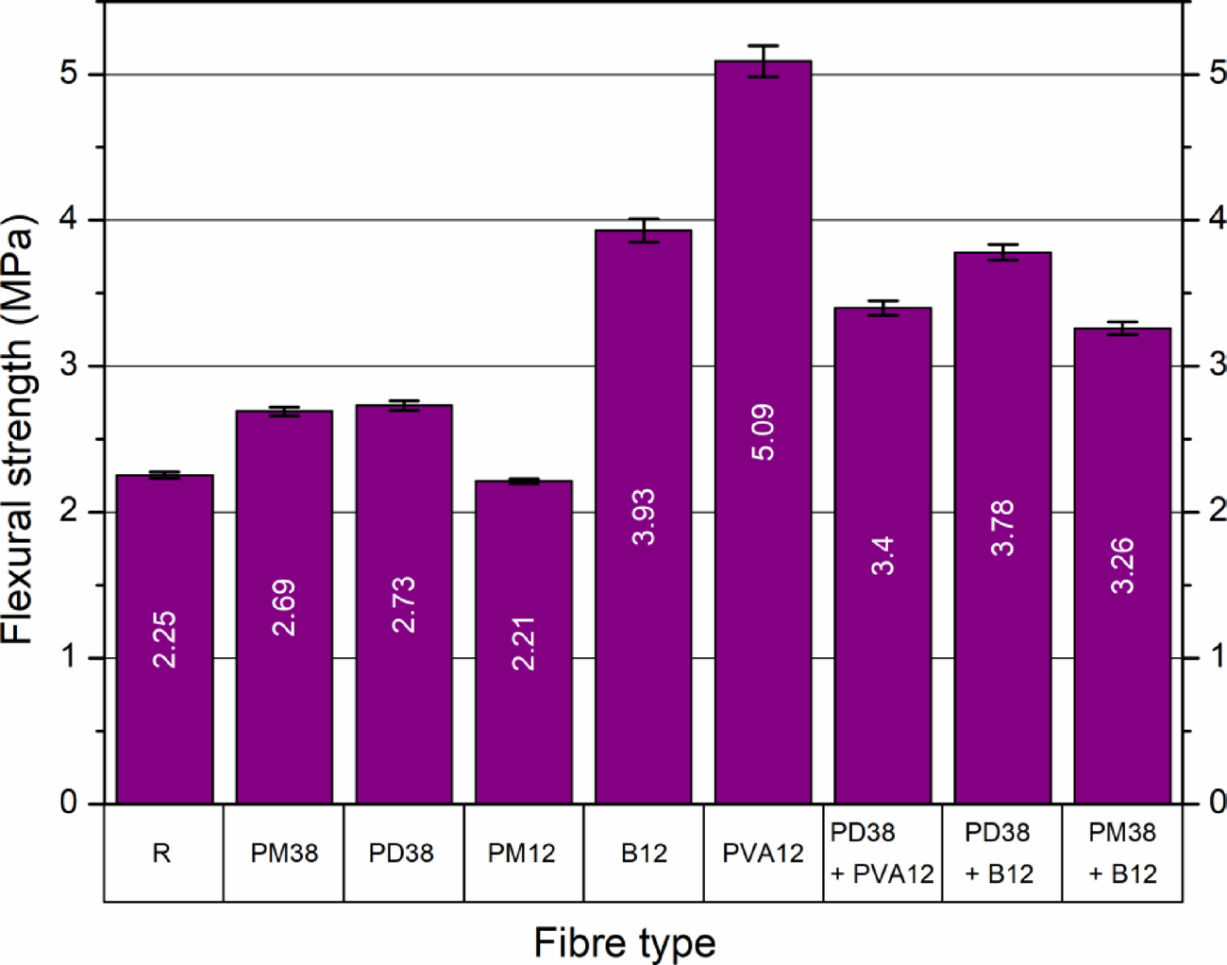
Flexural strength of HPSCC with different fibre types and combinations.

The flexural strength of the high-performance self-compacting concrete (HPSCC) beams, evaluated using a three-point bending test with an initial notch depth of 50 mm and an effective cross-sectional height of 90 mm, demonstrated significant variation depending on the type and configuration of fibres used (Fig. 12). The reference sample (R) reached a flexural strength of 2.25 MPa, a typical value expected for concrete matrices without fibre reinforcement. This finding aligns well with previous observations by Çelik and Bingöl^24,25^ and Feng et al.^22^, who indicated a substantially higher susceptibility to crack propagation and brittle fracture in fibre-free concrete.

Concrete mixes containing single types of polypropylene fibres, specifically PM38 and PD38, exhibited moderate, but noticeable, improvements in flexural strength compared to the plain concrete control. PM38 fibres, characterised by their mesh structure and 38 mm length, achieved a flexural strength of approximately 2.69 MPa. Similarly, concrete containing longer polypropylene crimped fibres (PD38) showed slightly higher performance, reaching a flexural strength of approximately 2.73 MPa. Both types of fibre provided a clear, albeit modest, increase relative to the control concrete (2.25 MPa). The observed improvement with PM38 fibres may be associated with their contribution to crack bridging after matrix cracking, thereby enhancing load transfer across the crack plane. This outcome aligns with the findings of Feng et al.^22^ and He et al.^55^, who documented incremental gains in flexural strength associated with polypropylene fibres. The slightly superior performance of PD38 fibres compared to PM38 is consistent with previous reports indicating the influence of fibre geometry and length on flexural response. This observation matches previous research by Smarzewski^56^ and Çelik and Bingöl^24,25^, highlighting that fibre length and stiffness critically influence the extent to which fibres can improve concrete flexural performance. These studies emphasise that longer fibres are typically more effective in bridging larger cracks, thus contributing more significantly to the overall mechanical behaviour of fibre-reinforced concrete.

The addition of short polypropylene fibres (PM12) showed minimal reinforcing effects, producing a strength of only 2.21 MPa. This limited effectiveness may be related to the reduced contribution of short fibres to crack bridging under flexural loading. This observation is consistent with the findings of He et al.^55^, who reported that lower content and shorter lengths of polypropylene fibres generally result in a relatively modest increase in tensile and flexural strength.

However, basalt fibres (B12) significantly improved the flexural strength to 3.93 MPa, which corresponds well to previous studies by Çelik and Bingöl^24,25^ and Smarzewski^56^. This improvement is consistent with the high stiffness and tensile capacity of basalt fibres reported in the literature. Polyvinyl alcohol (PVA) fibres exhibited the highest flexural strength among single fibre reinforced samples, achieving 5.09 MPa, representing an increase of approximately 121.7% compared to the control sample. This remarkable improvement is consistent with the results reported by Feng et al.^22^, highlighting the superior performance of PVA fibres as reported for fibre systems with strong fibre–matrix interaction and pronounced post-cracking response.

Hybrid fibre combinations demonstrated clear synergistic benefits. For example, the PD38 + PVA12 mixture, which contains longer polypropylene crimped fibres combined with shorter and well-bonded PVA fibres, reached a flexural strength of 3.40 MPa. This result indicates an enhanced flexural response compared to the single polypropylene fibre systems. Another hybrid combination, PM38 + B12, also showed a moderate improvement, achieving a strength of 3.26 MPa. The observed increase reflects the combined contribution of fibres with different stiffness and length. In particular, the hybrid combination PD38 + B12 achieved a significant flexural strength of 3.78 MPa, illustrating a substantial improvement consistent with previous studies by Çelik and Bingöl^24,25^ and Fu et al.^53^. The combined presence of basalt and crimped polypropylene fibres resulted in improved flexural performance under bending.

Interpreting these findings in the context of existing research indicates clearly that polymeric fibres (PVA), characterised by their high ductility and strong bond to the concrete matrix, significantly improve the mechanical properties of self-compacting concretes, particularly their flexural strength and post-cracking behaviour. Basalt fibres effectively enhance stiffness and load bearing capacity, especially when combined with long polypropylene fibres, further confirming previous research results. Hybrid fibre-reinforced mixtures were notably more effective than single-type fibre mixtures, a phenomenon well supported by Fu et al.^53^ and He et al.^55^, who highlighted the different reinforcement mechanisms activated by various types of fibre, resulting in complementary and enhanced performance. These results underscore the distinct advantages of hybrid fibre mixtures and polyvinyl alcohol (PVA) fibres over single-type polypropylene fibres in improving flexural strength. The presented findings carry practical implications for the design of high-performance concretes, emphasising the need to consider the synergistic effects of fibre blends with different lengths and mechanical properties.

#### Crack development and deformation behaviour at peak load

**Fig. 13:**
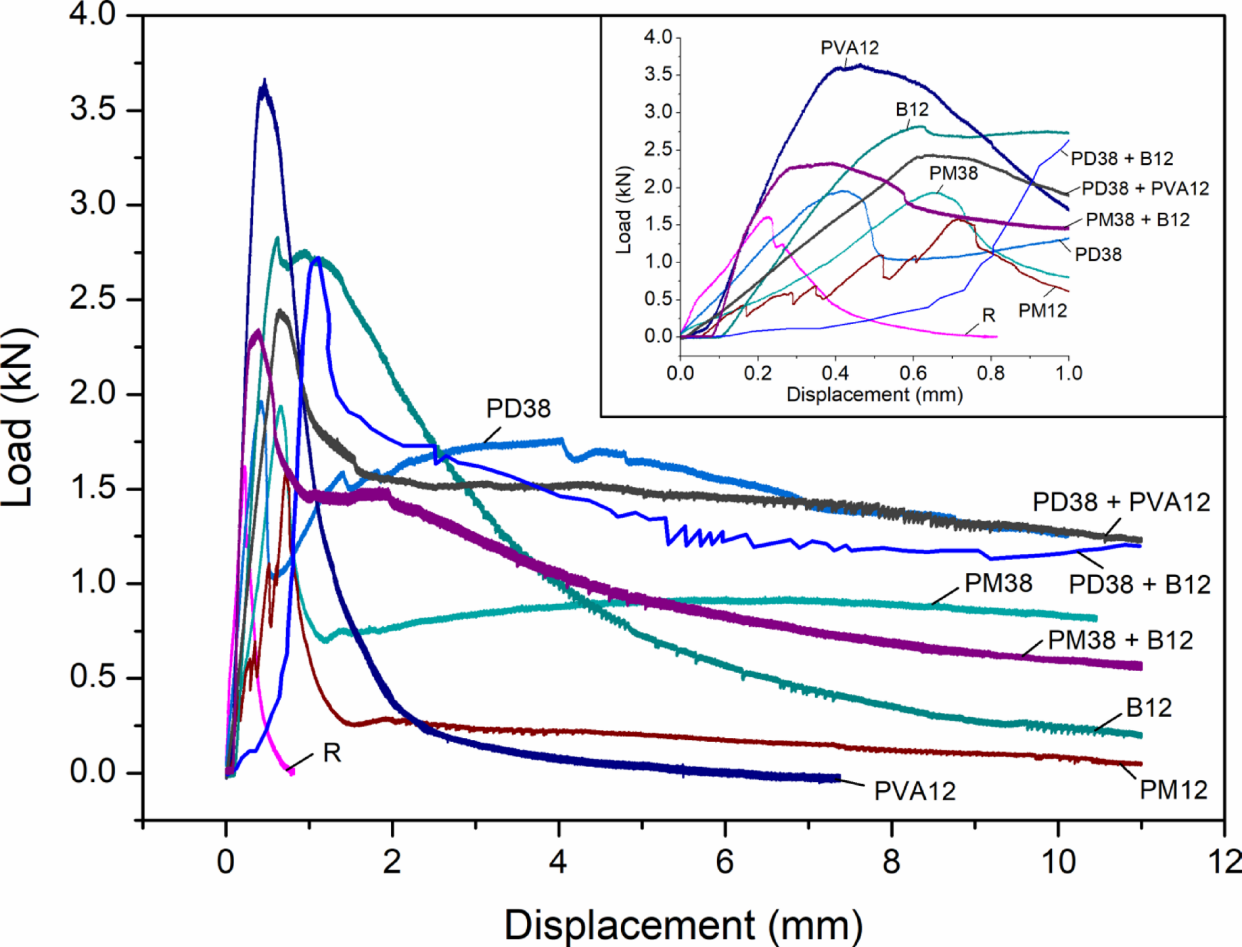
Load-displacement curves of HPSCC with different types of fibre and combinations.

**Fig. 14:**
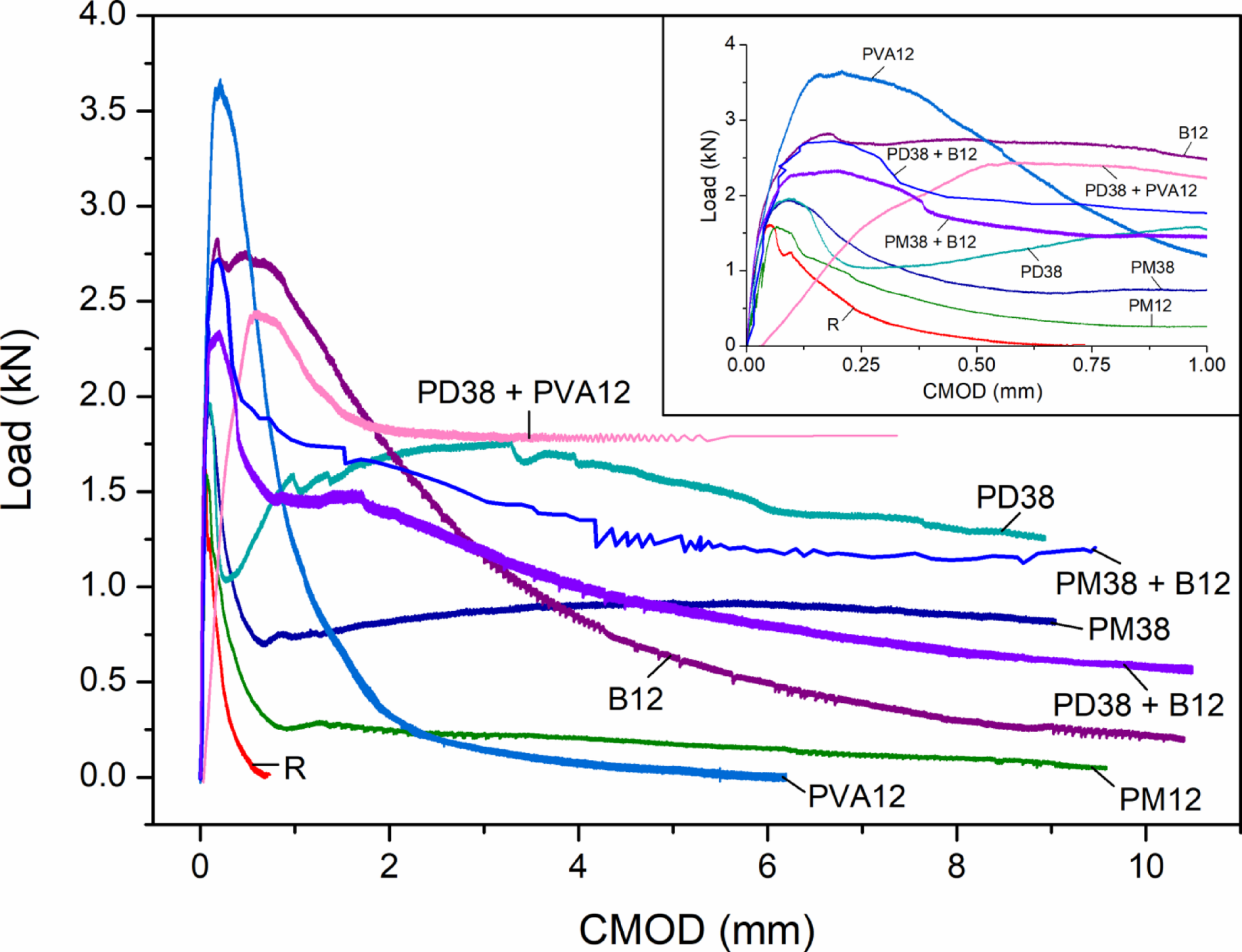
Load-CMOD curves of HPSCC with different fibre types and combinations.

Figures 13 and 14 present the global mechanical response of the nine concrete mixes under three-point bending, in terms of load–displacement and load–CMOD relationships, respectively. These curves highlight the brittle or ductile nature of each composition and provide insight into the macroscopic response of fibre-reinforced specimens during crack initiation and propagation.

The reference mix (R) showed typical brittle failure behaviour, with a steep increase in the load–displacement curve and a sharp post-peak drop, consistent with the abrupt propagation of a single dominant crack. The peak displacement reached only 0.22 mm, with a CMOD of 0.051 mm, corresponding to the formation of a singular macrocrack under peak stress. This behaviour aligns well with the observations reported by Çelik and Bingöl^24^ and Liang et al.^57^, where the control specimens without fibres exhibited the lowest deflection and narrowest crack width due to the absence of any bridging mechanism.

The addition of polypropylene fibres had a moderate effect on flexural behaviour. The PM12 mix showed a peak displacement of 0.72 mm, but a relatively low CMOD of 0.067 mm, indicating the development of multiple cracking prior to peak load. The PM38 and PD38 mixes exhibited displacements of 0.65 mm and 0.42 mm, with corresponding CMOD values of 0.085 mm and 0.095 mm, respectively. These results indicate an improved deformation capacity, but a tendency toward slightly larger crack openings compared to PM12. Smarzewski^5^, where increasing the polypropylene content improved ductility while maintaining moderate crack widths, reported similar trends. More substantial changes in cracking behaviour were observed with basalt fibres (B12) and polyvinyl alcohol (PVA12). The B12 mix achieved a higher peak load (2.83 kN) and a displacement of 0.62 mm, but with a CMOD of 0.180 mm, indicating a more pronounced macrocrack formation. This behaviour is consistent with the limited deformation capacity reported for stiff fibres in the literature^57^. The PVA12 mix reached the highest peak load (3.67 kN) and showed a displacement of 0.46 mm. However, it also exhibited the widest single crack (0.208 mm), reflecting a concentration of deformation in a dominant crack under peak load, as noted by Çelik and Bingöl^24^.

Hybrid fibre systems demonstrated the most diverse and informative behaviour. The PD38 + PVA12 mix exhibited a significant displacement of 0.65 mm, coupled with a large CMOD of 0.604 mm, indicating the formation of a dominant crack and substantial post-peak deformation. In contrast, PD38 + B12 showed both high ductility (1.11 mm) and a similarly wide crack (0.626 mm), highlighting the strong post-cracking deformation capacity of this hybrid system. Of particular note is the behaviour of PM38 + B12, which combined moderate displacement (0.39 mm) with a high CMOD (0.200 mm). This response indicates the development of a dominant crack despite the presence of two fibre types. These findings reinforce the conclusion that the mechanical synergy between long and short fibres, as well as their orientation and interaction with the matrix, is critical in controlling crack formation and distribution.

**Fig. 15:**
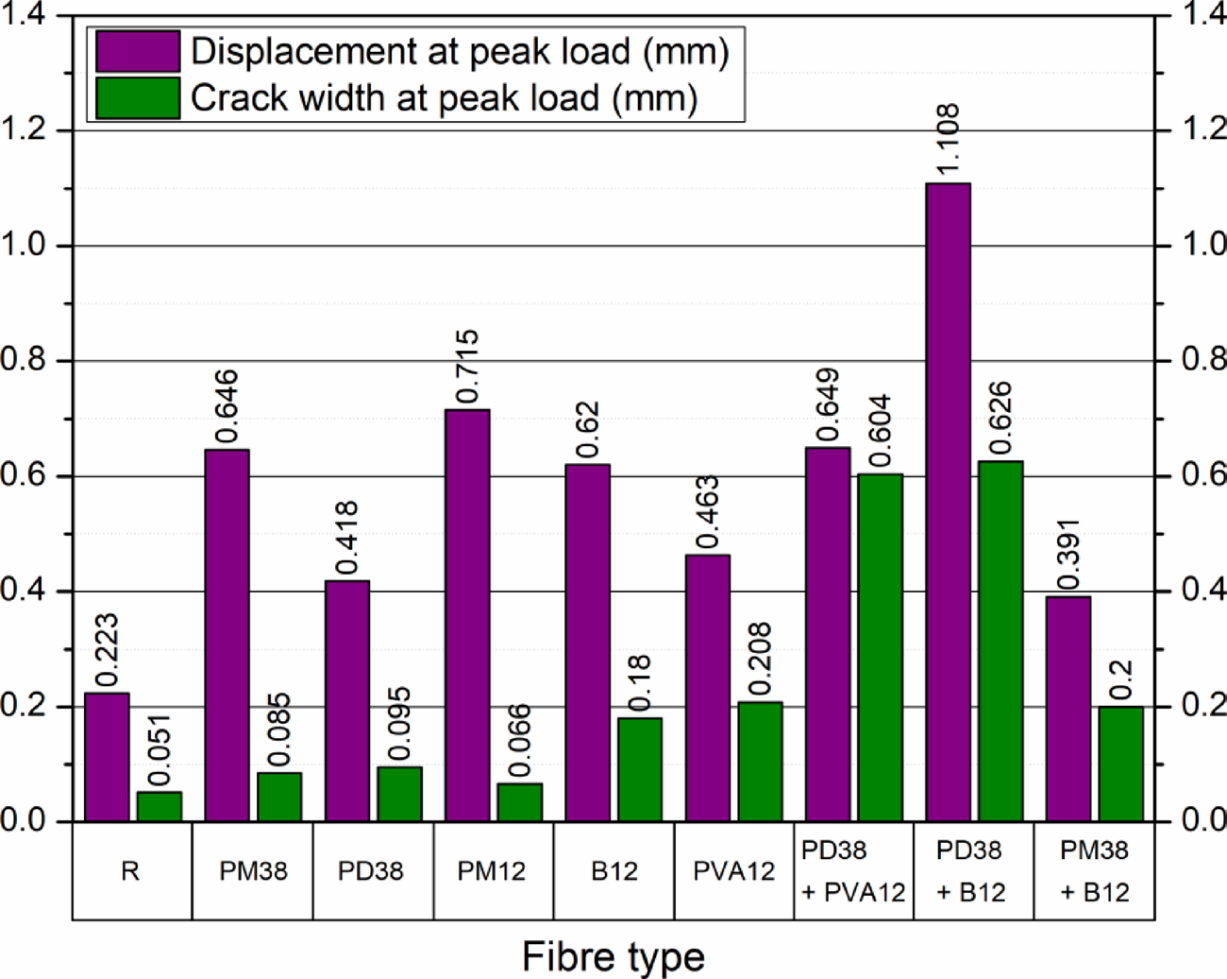
Displacement and crack width at peak load with different fibre types and combinations.

Figure 15 summarises these effects by comparing displacement and CMOD values at peak load. A general trend of increasing CMOD with increasing fibre stiffness or bonding strength can be observed, particularly in PVA- and basalt-reinforced systems. However, mixes such as PM12 and PD38 managed to limit crack width while still improving displacement, showing promise for applications requiring both durability and serviceability.

In general, all fibre-reinforced mixes outperformed the control in terms of both load capacity and ductility. However, the type, length, and combination of fibres substantially affected crack morphology. These findings support the conclusion that fibre hybridisation can enhance flexural performance, while the control of crack width remains strongly dependent on fibre type and mixture composition.

#### Fracture energy, toughness and reinforcement efficiency of fibre-reinforced HPSCC

**Fig. 16:**
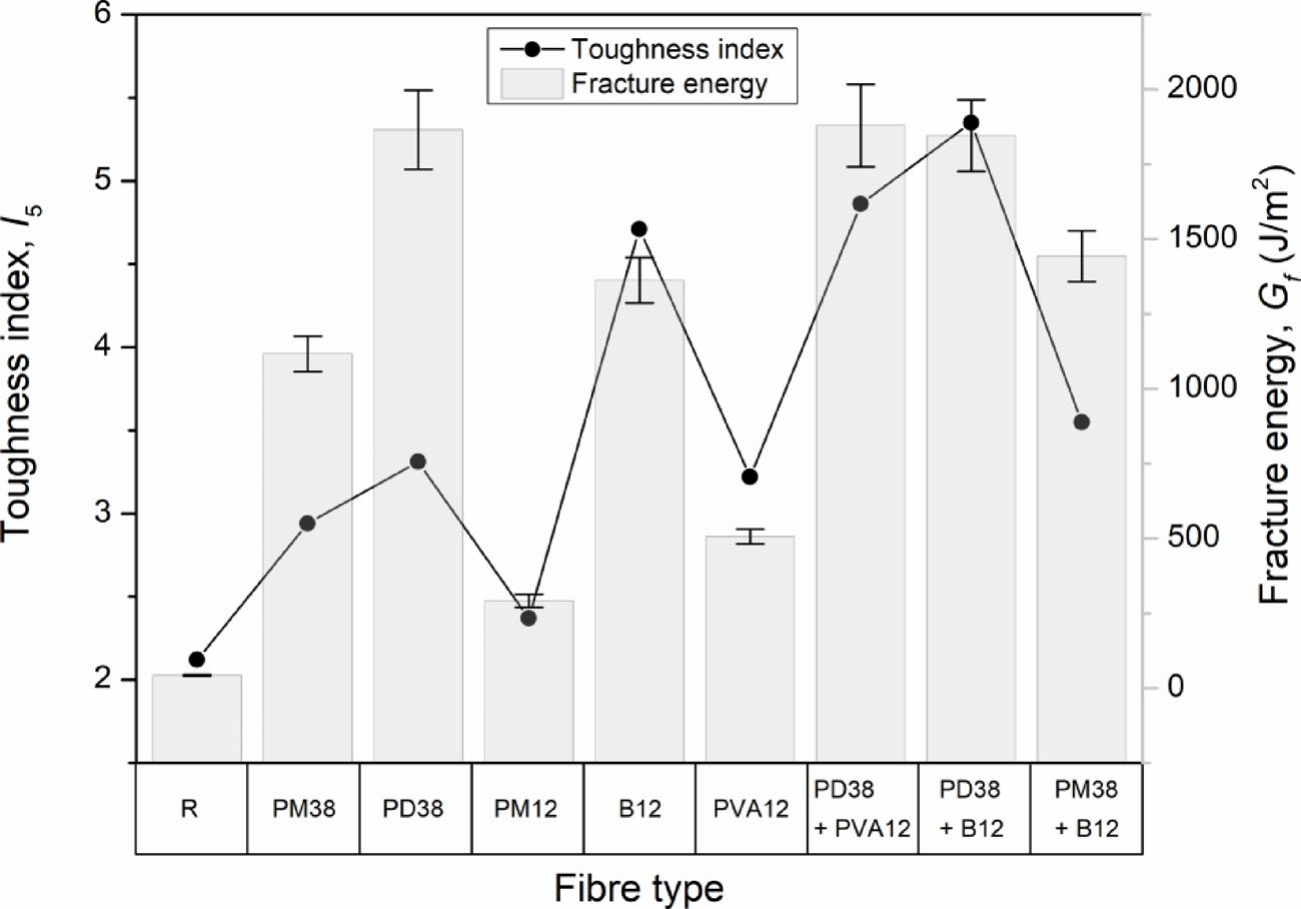
Fracture energy *G*_*f*_ and toughness index *I*_5_ of HPSCC with different fibre types.

To evaluate the fracture resistance of high-performance self-compacting concrete (HPSCC), a comprehensive assessment was performed based on the area under the load–CMOD curve (fracture energy, *G*_*f*_), toughness indices according to ASTM C1609/C1609M-19^58^, and reinforcement efficiency coefficients. The complete results are presented in Table 9 and visualised in Fig. 16.

**Table 9:** Parameters of fracture energy.

Fibre type	G_f_ (J/m^2^)	I_5_	I_10_	η_Gf_	η_I5_
R	43.2	2.12	1.6	1.0	1.0
PM38	1116.3	2.94	5.2	25.84	1.38
PD38	1864.9	3.31	6.83	43.17	1.56
PM12	291.7	2.37	3.28	6.75	1.12
B12	1362.7	4.71	7.71	31.54	2.22
PVA12	506.4	3.22	3.83	11.72	1.52
PD38 + PVA12	1879.1	4.86	15.53	43.5	2.29
PD38 + B12	1845.4	5.35	9.52	42.72	2.53
PM38 + B12	1443.3	3.55	6.19	33.41	1.67

All mixes containing fibres significantly improved the fracture energy compared to the reference concrete (R), which only recorded 43.2 J/m². This increase is associated with the presence of fibres, which alter the crack propagation process and enable higher energy absorption prior to failure. As explained by Li et al.^59^, fibres with varying stiffness and elongation capacities act synergistically to limit macrocrack growth and crack opening width.

Among concretes with a single fibre type, the best performance was observed for PD38 (43.17× improvement in Gf) and B12 (31.54×). These results indicate a strong contribution of these fibres to the fracture resistance of the composite. Similar trends were documented by Çelik and Bingöl^24^, where 12 mm basalt fibres increased the fracture energy from 0.18 to 0.29 N/mm, while polypropylene fibres achieved higher values (up to 0.75 N/mm), due to their high elongation (~ 25%) and pull-out capacity. Likewise, Smarzewski^5^ reported that the fracture energy increased from 0.03 N/mm (reference) to 0.77 N/mm for polypropylene and 0.29 N/mm for additions of basalt fibres, with hybrid mixes ranging from 0.84 N/mm.

The influence of short fibres (PM12) was limited in comparison (6.75× increase), suggesting a reduced contribution to fracture energy under bending for this fibre length. The toughness indices 5 = 2.37 and 10 = 3.2 reflect a moderate post-peak behaviour. The PVA fibres (PVA12) offered a balanced improvement in fracture energy (506.4 J/m²) and toughness (_5_ = 3.22). Their contribution to post-peak behaviour was intermediate compared to polypropylene-based and hybrid systems. As noted by Çelik and Bingöl^25^, the fibre failure mode influences post-cracking behaviour, with polypropylene fibres typically exhibiting pull-out and basalt fibres showing more limited deformation prior to failure.

The enhanced fracture energy observed in mixtures containing PVA fibres, particularly in hybrid systems, can be discussed in the context of fibre–matrix interaction reported in the literature. Previous microstructural studies have demonstrated that PVA fibres exhibit a dense and continuous interfacial transition zone with cementitious matrices, characterised by strong physico-chemical bonding and reduced porosity^60,61^. The hydrophilic nature of PVA fibres promotes improved wetting and local densification of the matrix, which enhances stress transfer at early stages of cracking. In the present study, no microstructural analyses were performed. Therefore, these mechanisms are discussed by analogy with established findings rather than directly confirmed.

Hybridisation of fibres led to the highest enhancements in both energy absorption and ductility. Among the combinations tested, the mix with the PD38 and PVA12 fibres achieved the best performance, reaching a fracture energy of 1879.1 J/m² and a remarkably high toughness index 10 = 15.53, indicating an enhanced post-cracking deformation capacity. Similarly, the hybrid mix containing PD38 and basalt fibres (B12) yielded a fracture energy of 1845.4 J/m², with an impressive toughness index _5_ = 5.35, demonstrating a high level of energy absorption after peak load. The PM38 + B12 mix also demonstrated significant energy absorption, achieving 1443.3 J/m², although the enhancement was less pronounced than for the PD38-based hybrids. The observed trends are consistent with the findings of Soe et al.^62^, who stated that hybrid fibres, which differ in length, stiffness, or strength, enhance crack resistance more effectively than single-type systems. Long fibres control macrocracks, while short fibres limit microcrack initiation and propagation. As further confirmed by Arslan^63^, even 0.1% basalt fibre increased fracture energy by 31.2%, highlighting the benefits of strategic fibre inclusion.

Figure 16 presents the fracture energy and corresponding toughness indices 5 for all HPSCC mixes tested with fibre reinforced HPSCCs. The data reveal a consistent trend in which hybrid fibre systems demonstrate superior performance over single-fibre mixes in terms of both energy absorption and post-cracking ductility. Among all combinations, systems based on PD38 fibres yielded the most favourable results, particularly when incorporated into hybrid mixes. In contrast, mixes containing only short or low-modulus fibres, such as PM12 or PVA12, exhibited more moderate enhancements.

The effectiveness of fibre reinforcement can be further evaluated using dimensionless reinforcement efficiency factors, defined by the following relationships:

4 $${\eta _{{G_f}}}=\frac{{{G_{f,{\mathrm{fibre}}}}}}{{{G_{f,{\mathrm{ref}}}}}},~ {\eta _{{I_5}}}=\frac{{{I_{5,{\mathrm{fibre}}}}}}{{{I_{5,{\mathrm{ref}}}}}}$$

These ratios, summarised in Table 9, quantify the degree to which a given type or combination of fibres improves the ability of concrete to resist fracture and sustain load after cracking. Notably, the reinforcement efficiency *η*_*Gf*_ exceeded 40 for the PD38 mix and its hybrids with B12 and PVA12 fibres, indicating a substantial enhancement in fracture-related performance compared to the reference concrete.

**Fig. 17:**
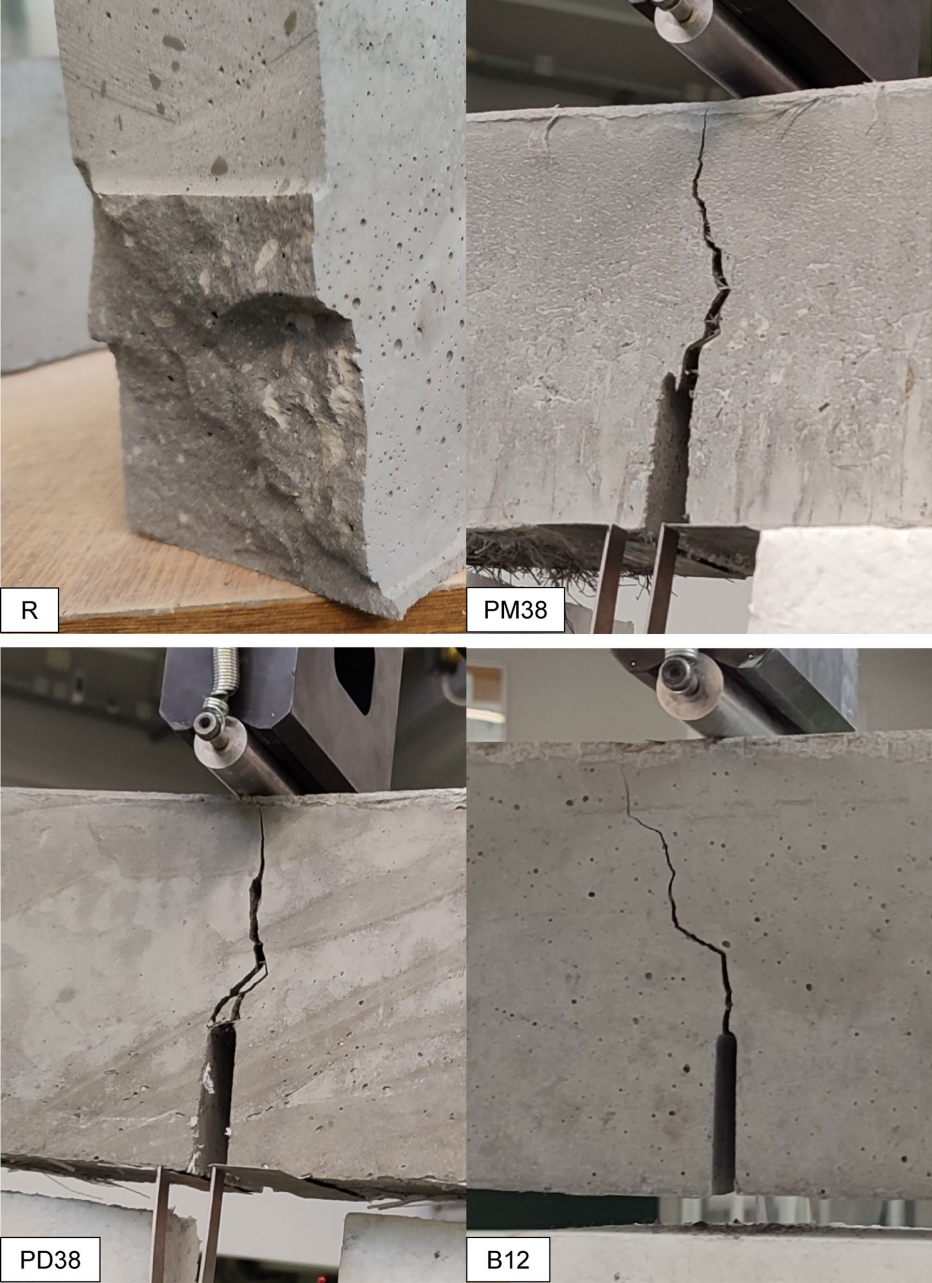

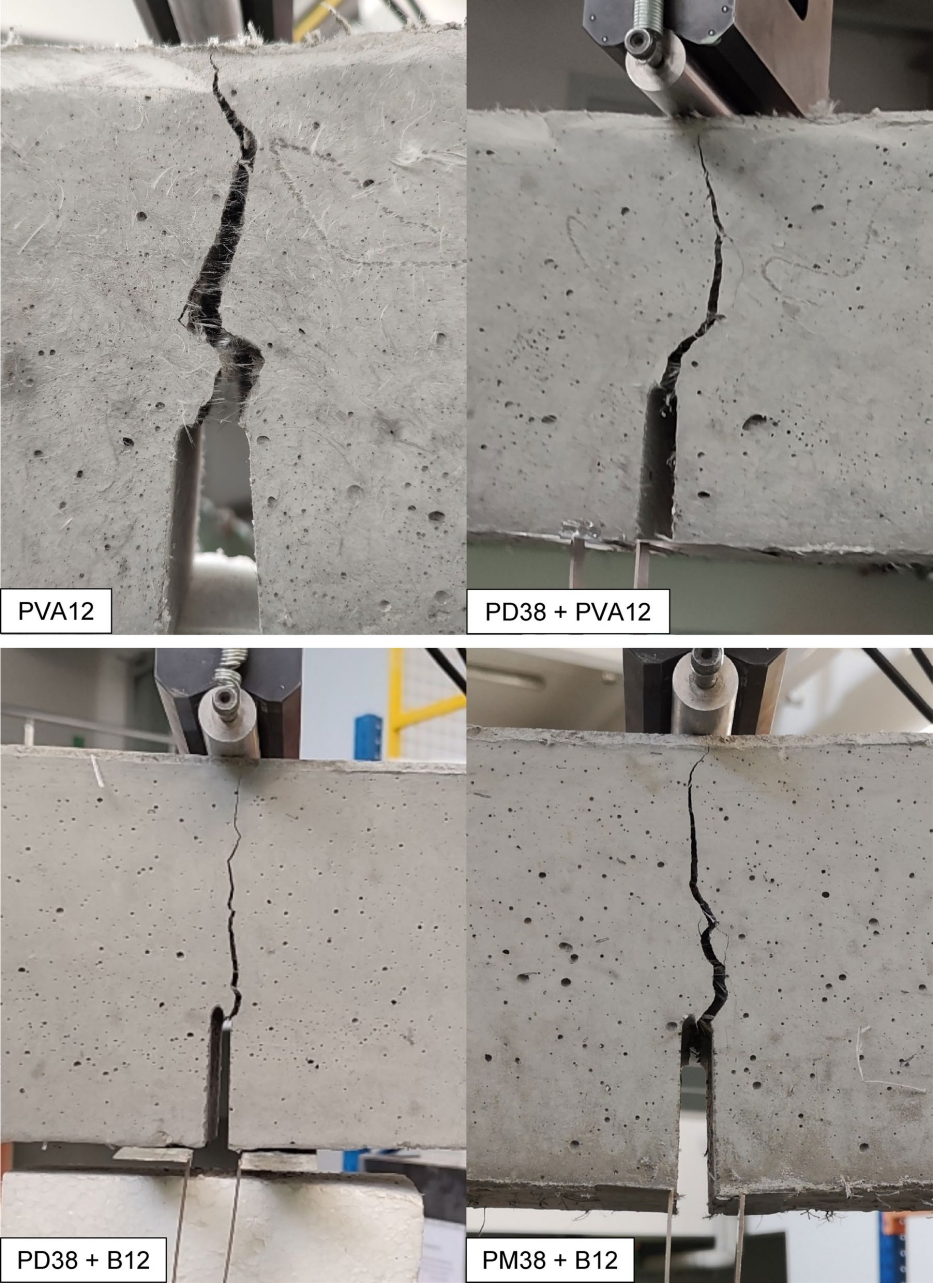
Failure modes of HPSCC samples reinforced with various types of fibres under bending. The sample series are identified in the lower left corner of each image.

Based on the visual inspection of the fracture surfaces shown in Fig. 17, the failure mechanisms of the fibre-reinforced HPSCC mixes vary significantly with the type and configuration of the fibre. The reference sample (R), which contained no fibres, exhibited a single planar macrocrack propagating vertically from the notch. The fracture surface is relatively smooth, showing little to no roughness or branching, which is typical of a matrix-dominated, brittle failure. The absence of bridging elements, such as fibres or aggregate interlock, resulted in an abrupt loss of load carrying capacity and minimal energy dissipation, as previously described for plain concrete by Çelik and Bingöl^24,25^ and Smarzewski^5^.

The dominant crack is straight and relatively smooth, but the fracture surface reveals moderate roughness and micro-chipping. Isolated remnants of fibres are visible along the crack plane, indicating limited interaction at the macroscopic scale. The fibres appear to have been displaced rather than fractured, suggesting a limited contribution to crack bridging under bending. No branching is observed, and the crack path remains unidirectional, indicating slightly enhanced but still brittle behaviour compared to the reference.

In stark contrast, the introduction of fibres fundamentally altered the fracture morphology and post-cracking behaviour. For the PM38 mix, a single dominant crack still formed from the notch, but the fracture surface showed pronounced roughness and a tortuous path, with a noticeable lateral kink near the top. Numerous long polymer fibres were observed that bridging the crack, spanning its width and resisting further opening. Several long polymer fibres were observed bridging the crack opening. The fibre ends appeared irregular, consistent with fibre pull-out or partial debonding rather than clean rupture. The presence of fibre bridging was accompanied by increased deformation capacity after first cracking, in agreement with trends outlined by Li et al.^59^. Similarly, the PD38 mix exhibited a central crack with a slightly jagged path and moderate opening width. The surface of the fracture showed the presence of protruding fibres, some partially embedded and others exposed as short strands along the crack. These features indicate fibre participation in crack bridging and post-cracking load transfer, although the overall response remained semi-ductile.

The B12 specimen, reinforced with short basalt fibres, presented a straight and relatively narrow crack with minimal roughness. Fibre residues were rarely observed on the fracture surface, indicating a limited macroscopic contribution of the fibres to crack bridging. The fracture morphology closely resembled that of the reference mix, suggesting that short, stiff fibres were less effective in bridging the crack and improving post-crack energy absorption, as also noted by Arslan^63^. In the PVA12 mix, the main crack followed a direct path, but the fracture surface was distinctly rough and covered with a dense network of fine white PVA fibres. These fibres formed a visible network across the crack plane, demonstrating extensive fibre engagement during crack opening. The absence of clearly ruptured fibre ends and the presence of numerous anchored fibres suggest a strong fibre–matrix interaction, with deformation accommodated through fibre stretching and pull-out. As a result, the failure mode exhibited pronounced ductility, consistent with the high toughness indices^63^.

Hybrid fibre systems, such as PD38 + PVA12 and PD38 + B12, showed the most complex fracture morphologies. In the PD38 + PVA12 specimen, the main crack was irregular and followed a zigzag path through the depth of the beam. Both PVA and PD fibres were evident across the fracture surface, many spanning the crack and others protruding from the matrix. Macroscopic features such as fibre pull-out marks and increased surface roughness indicate that both fibre types contributed to crack resistance and post-cracking deformation. The PD38 + B12 mix showed a similarly irregular fracture surface, with visible fibre-related features and interfacial separation. The presence of two fibre types promoted crack deflection and enhanced energy dissipation, resulting in a semi-ductile failure response.

The PM38 + B12 sample exhibited a slightly rough and tortuous crack, with several long fibres bridging the opening and short basalt fibres visible as fine remnants along the faces. At the macroscopic scale, the fracture behaviour was dominated by the contribution of long polymer fibres, while the basalt fibres appeared to contribute primarily at early stages of crack formation.

In summary, photographic evidence demonstrates that the addition of fibres (particularly long or hybrid types) transforms the fracture behaviour of HPSCC from brittle and matrix controlled failure to a much tougher and more ductile regime. This transformation is associated with increased fibre engagement, crack deflection and post-cracking deformation observed at the specimen scale. The degree of energy dissipation and crack width control depend on the type, length, and surface characteristics of the fibres, with hybrid systems showing the greatest synergy and improvement. These observations are consistent with the findings of Çelik and Bingöl^24,25^, Smarzewski^5^, Arslan^63^, and Wang et al.^64^.

**Fig. 18:**
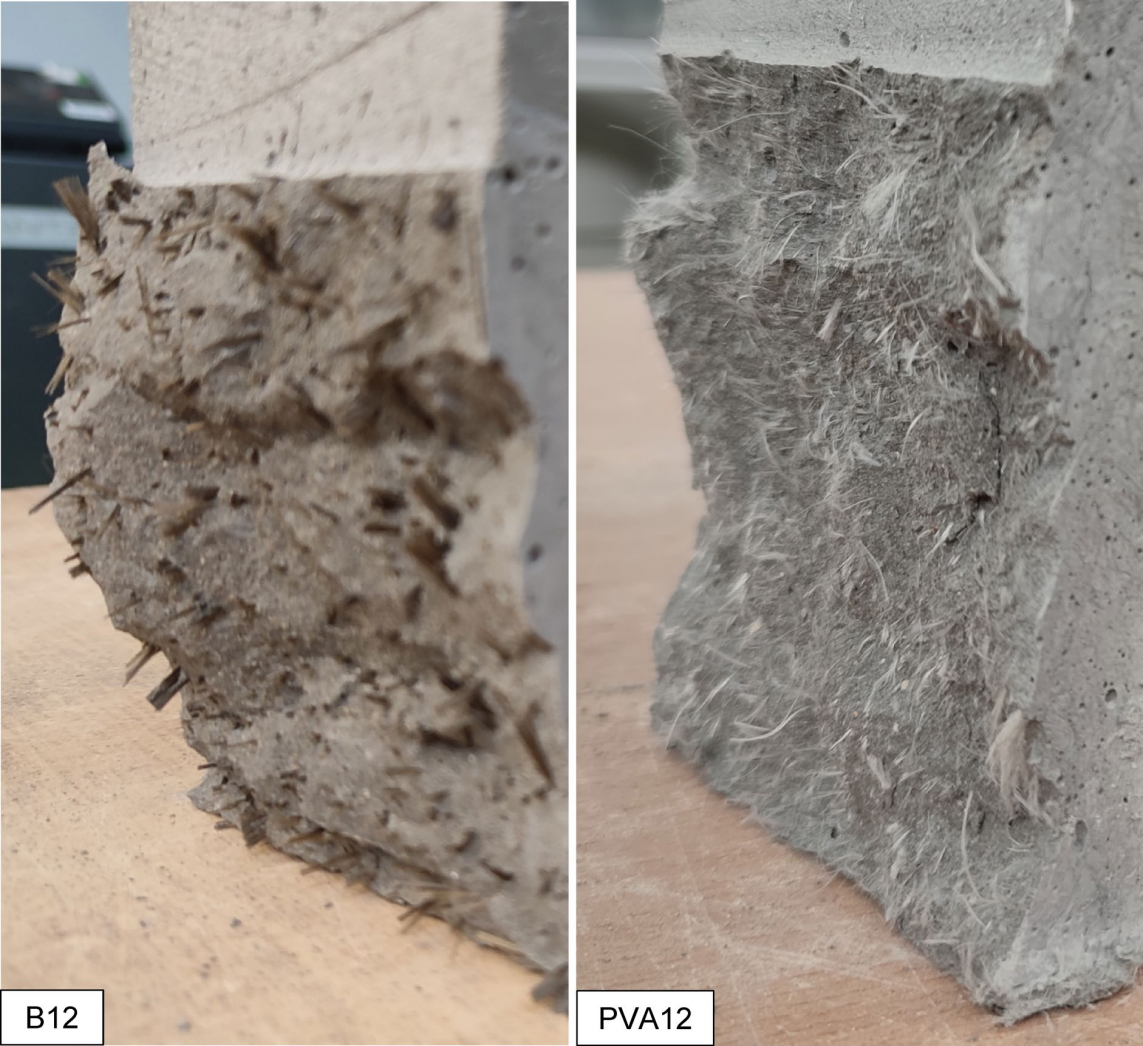
Representative fracture surfaces of HPSCC specimens reinforced with short basalt fibres (B12) and polyvinyl alcohol fibres (PVA12) after flexural testing.

To further illustrate the influence of fibre type on fracture morphology under bending, representative fracture surfaces of specimens reinforced with short basalt fibres (B12) and PVA fibres (PVA12) are shown in Fig. 18, complementing the overview of failure modes presented in Fig. 17.

In the B12-reinforced specimens, basalt fibres were not prominently visible in the central part of the fracture surface, while individual fibres could be identified mainly near the specimen edges (Fig. 18). At the macroscopic scale, this indicates a limited contribution of short basalt fibres to bridging of the dominant macro-crack, with fibre engagement occurring primarily in localised zones. The absence of extensive fibre pull-out and the relatively compact fracture surface are consistent with the high stiffness and brittle nature of basalt fibres, which tend to fracture or debond at relatively small crack openings.

In contrast, the PVA12-reinforced specimens exhibited a distinctly fibrous fracture surface, with short PVA fibres clearly visible across the entire cross-section (see Fig. 18). While isolated fibre pull-out was locally observed near the specimen edges, the majority of PVA fibres appeared to be ruptured, particularly in the central part of the fracture surface. This indicates that crack bridging was dominated by fibre stretching and rupture rather than extraction. At the macroscopic scale, this behaviour is consistent with the strong fibre–matrix interaction of PVA fibres reported in the literature and provides a qualitative explanation for the enhanced fracture energy and distributed cracking observed for this mixture.

#### Hybrid effect on fracture energy

**Fig. 19:**
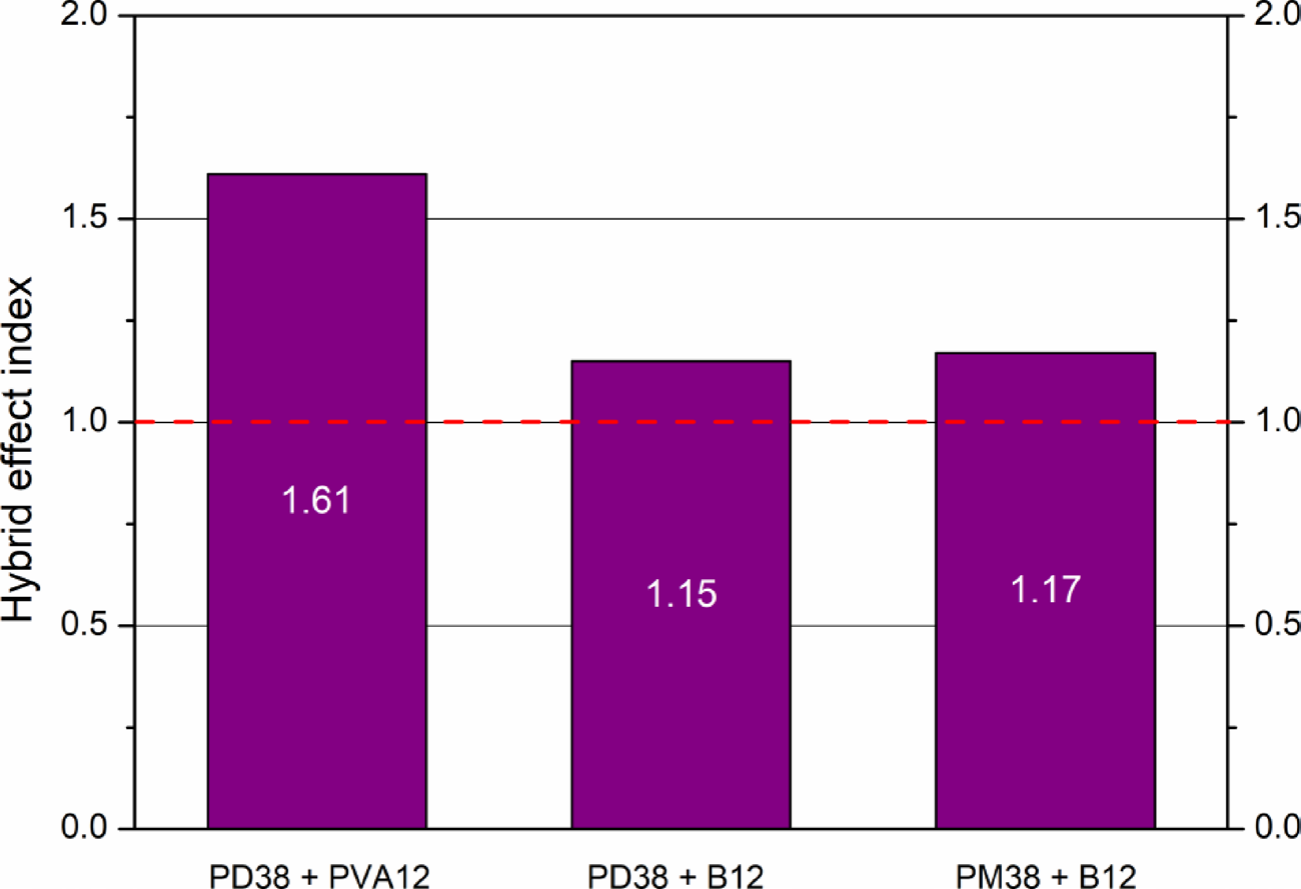
Hybrid effect index for different fibre combinations.

The hybrid synergistic effect on fracture energy was evaluated for all hybrid fibre systems using the hybrid index (α), as per Eq. (3). The calculated indices, based on the values in Table 9, clearly indicate a positive hybrid effect for each combination, with all *α* values exceeding unity (Fig. 19).

The highest synergy was observed for the hybrid PD38 + PVA12, with α = 1.61, indicating that this system outperformed the weighted average of its single fibre concretes by more than 60%. This pronounced hybrid effect is associated with the simultaneous presence of long crimped polypropylene macrofibres (PD38) and short PVA microfibres, which act at different stages of crack development. The PD38 fibres contribute to crack bridging and load transfer at larger crack openings, while the PVA fibres participate in the control of microcrack evolution and post-peak response. The combined action of fibres with different lengths and bonding characteristics results in enhanced energy absorption and ductility, in agreement with the observations of Soe et al.^62^ and Song and Yin^44^.

Both the hybrids PD38 + B12 (α = 1.15) and PM38 + B12 (α = 1.17) also showed a clear positive effect, confirming that combinations of rigid basalt fibres with flexible macro-polypropylene or polyolefin fibres can lead to performance gains that exceed what would be expected from their individual contributions. Although the magnitude of the hybrid effect was lower than that observed for PD38 + PVA12, it remained significant, and these hybrids yielded much greater fracture energy than any single-fibre system alone. This observation highlights the role of fibre complementarity in terms of stiffness, length and interaction with the matrix, which governs crack control across multiple scales.

In summary, the positive hybrid indices for all investigated systems demonstrate that synergistic effects on fracture behaviour are achievable in HPSCC through rational hybridisation of fibres. These results not only validate previous findings reported by Feng et al.^22^, Song and Yin^44^, and Soe et al.^62^, but also provide a quantitative framework for comparing hybrid fibre efficiency based on fracture-energy-related metrics, supporting the optimisation of fibre combinations in advanced concrete design.

Across all investigated mixtures, clear qualitative interconnections can be identified between fresh-state behaviour, mechanical performance and fracture response. Fibre systems that caused pronounced increases in viscosity and reduced passing ability (notably PVA-rich Across all investigated mixtures, clear qualitative interconnections can be identified between fresh-state behaviour, mechanical performance and fracture response. Fibre systems that caused pronounced increases in viscosity and reduced passing ability (notably PVA-rich fibre systems) generally exhibited enhanced crack-bridging efficiency and higher fracture energy, indicating a trade-off between rheological performance and post-cracking resistance. Conversely, mixtures that preserved favourable flowability and passing ability, such as those reinforced with long polymer fibres or polymer–basalt hybrids, showed more balanced mechanical performance with moderate improvements in strength and fracture toughness.

These trends suggest that fresh-state rheology governs fibre dispersion and orientation, which in turn influences crack initiation, propagation and energy dissipation under mechanical loading. Although no direct statistical correlations were established, the consistent alignment of rheological, mechanical and fracture-related responses across the test series supports the interpretation that fibre type, length and hybridisation act as unifying parameters linking workability, strength development and fracture behaviour in HPSCC.

## Conclusions

This study examined the influence of non-metallic fibres (polyolefin, polypropylene, basalt and polyvinyl alcohol), used individually and in hybrid systems, on the fresh-state performance, mechanical, and fracture behaviour of high-performance self-compacting concrete (HPSCC). Based on the experimental results, the following key conclusions can be drawn:

1. Long crimped polypropylene fibres (PD38) provided the most pronounced improvement in compressive and splitting tensile strength, whereas long extruded polyolefin fibres (PM38) were less effective, likely due to reduced dispersion efficiency.
2. Short, stiff fibres (PVA12 and basalt B12) produced substantially higher flexural strength and more stable post-peak behaviour than polyolefin fibres, highlighting their effectiveness in crack control at small crack openings.
3. Hybrid systems, particularly PD38 + PVA12 and PD38 + B12, exhibited the highest fracture energy and residual load-carrying capacity, demonstrating that combining long ductile fibres with short stiff fibres enhances energy dissipation beyond that of single-fibre systems.
4. Fibre-reinforced mixtures showed sustained post-peak response and increased crack-opening tolerance, with the most pronounced effects observed in PD38-based hybrid mixes.
5. Mixtures containing short and fine fibres, especially PVA12, exhibited significant reductions in passing ability and flowability, whereas long polyolefin and basalt fibres had a less detrimental effect on self-compactability.
6. While PD38 + PVA12 maximised fracture energy, it suffered from severe workability loss, whereas PD38 + B12 provided a more balanced combination of strength, toughness and fresh-state performance.
7. From a practical perspective, the results indicate that tailored hybrid fibre systems can be effectively used to enhance the ductility and fracture resistance of HPSCC, particularly for thin-walled elements, complex formworks or densely reinforced members where conventional compaction is impractical.
8. A limitation of the present study is the absence of microscopic characterisation and direct fibre–matrix bond testing. Therefore, mechanistic interpretations are presented as literature-supported inferences consistent with the observed mechanical response. Future studies should focus on microstructural investigations and optimisation of fibre proportions and admixture systems to mitigate the workability–toughness trade-off.

## Data Availability

All data generated or analysed during this study are available from the corresponding author upon reasonable request.

## References

[1] Scrivener, K. L., John, V. M. & Gartner, E. M. Eco-efficient cements: Potential economically viable solutions for a low-CO₂ cement-based materials industry. *Cem. Concr. Res.* **114**, 2–26. 10.1016/j.cemconres.2018.03.015 (2018).

[2] Neville, A. M. *Properties of Concrete* 5th ed. (Pearson Education Limited, 2011).

[3] Pająk, M. & Ponikiewski, T. Experimental investigation on hybrid steel fibers reinforced self-compacting concrete under flexure. *Procedia Eng.* **193**, 218–225 (2017).

[4] Smarzewski, P. & Jancy, A. Comparative study on mechanical performance and toughness of high-performance self-compacting concrete with polypropylene and basalt fibres. *Materials* **18**, 3833 (2025). 40870150 10.3390/ma18163833 PMC12387742

[5] Smarzewski, P. Influence of basalt-polypropylene fibres on fracture properties of high performance concrete. *Compos. Struct.* **209**, 23–33 (2019).

[6] Kim, D. J., Naaman, A. & El-Tawil, S. Comparative flexural behavior of four fiber reinforced cementitious composites. *Cem. Concr. Compos.* **30**, 917–928 (2008).

[7] Kuder, K. & Shah, S. Processing of high-performance fiber-reinforced cement-based composites. *Constr. Build. Mater.* **24**, 181–186 (2010).

[8] Branston, J., Das, S., Kenno, S. & Taylor, C. Mechanical behaviour of basalt fibre reinforced concrete. *Constr. Build. Mater.* **124**, 878–886 (2016).

[9] Yoo, D.-Y. & Banthia, N. Impact resistance of fiber-reinforced concrete – A review. *Cem. Concr. Compos.* **104**, 103389 (2019).

[10] Brandt, A. Fibre reinforced cement-based (FRC) composites after over 40 years of development in building and civil engineering. *Compos. Struct.* **86**, 3–9 (2008).

[11] Algin, Z. & Ozen, M. The properties of chopped basalt fibre reinforced self-compacting concrete. *Constr. Build. Mater.* **186**, 678–685 (2018).

[12] Rooholamini, H., Hassani, A. & Aliha, M. R. M. Fracture properties of hybrid fibre-reinforced roller-compacted concrete in mode I with consideration of possible kinked crack. *Constr. Build. Mater.* **187**, 248–256 (2018).

[13] Wang, D., Ju, Y., Shen, H. & Xu, L. Mechanical properties of high performance concrete reinforced with basalt fiber and polypropylene fiber. *Constr. Build. Mater.* **197**, 464–473 (2019).

[14] Banthia, N., Majdzadeh, F., Wu, J. & Bindiganavile, V. Fiber synergy in hybrid fiber reinforced concrete (HyFRC) in flexure and direct shear. *Cem. Concr. Compos.* **48**, 91–97 (2013).

[15] Wang, J., Wang, Y. & Li, Z. Synergistic effects of hybrid fibres on the mechanical performance and durability of ultra-high performance concrete: A review. *Cem. Concr. Compos.* **139**, 104885. 10.1016/j.cemconcomp.2023.104885 (2023).

[16] Błaszczyk, K. & Smarzewski, P. Influence of hybrid fibers on workability, mechanical and dynamic properties of ultra-high performance concrete. *Appl. Sci.* **15**, 5716 (2025).

[17] Dziomdziora, P. & Smarzewski, P. Effect of hybrid fiber compositions on mechanical properties and durability of ultra-high-performance concrete: A comprehensive review. *Materials* **18**, 2426 (2025). 40508423 10.3390/ma18112426 PMC12156361

[18] Gołaszewski, J. Technology of self-compacting concrete versus traditionally compacted concrete, Przegląd Budowlany, vol. 6, (in Polish) (2009).

[19] Jasiczak, J., Wdowska, A. & Rudnicki, T. Ultra-high performance concretes: properties, technology, applications, 2008. (in Polish).

[20] Yoo, D.-Y., Shin, H., Yang, J.-M. & Yoon, Y.-S. Material and bond properties of ultra high performance fiber reinforced concrete with micro steel fibers. *Compos. Part B Eng.* **58**, 122–133 (2014).

[21] Guerini, V., Conforti, A., Plizzari, G. & Kawashima, S. Influence of steel and macro-synthetic fibers on concrete properties. *Fibers* **6**, 47 (2018).

[22] Feng, J. et al. Experimental study on hybrid effect evaluation of fiber reinforced concrete subjected to drop weight impacts. *Materials* **11**, 2563 (2018). 30562942 10.3390/ma11122563 PMC6315794

[23] Mazaheripour, H., Ghanbarpour, S., Mirmoradi, S. & Hosseinpour, I. The effect of polypropylene fibers on the properties of fresh and hardened lightweight self-compacting concrete. *Constr. Build. Mater.* **25**, 351–358 (2011).

[24] Çelik, Z. & Bingöl, A. F. Fracture properties and impact resistance of self-compacting fiber reinforced concrete (SCFRC). *Mater. Struct.*10.1617/s11527-020-01487-8 (2020).

[25] Çelik, Z. & Bingöl, A. F. Mechanical properties and postcracking behavior of self-compacting fiber reinforced concrete. *Struct. Concr.*10.1002/suco.201900396 (2019).

[26] Liu, X., Wu, T., Yang, X. & Wei, H. Properties of self-compacting lightweight concrete reinforced with steel and polypropylene fibers. *Constr. Build. Mater.* **226**, 388–398 (2019).

[27] Augustino, D., Onchiri, R., Kabubo, C. & Kanali, C. Mechanical and durability performance of high-strength concrete with waste tyre steel fibres. *Adv. Civil Eng.*10.1155/2022/4691972 (2022).

[28] Ramalingam, V., Vaishnavi, M. & Geetha, R. Study on the workability, mechanical properties of fish tail palm fibre reinforced concrete-emphasis on fibre content and fibre length. *Eur. J. Environ. Civ. Eng.* **27**, 1–19 (2022).

[29] PN-EN 197-1:2012. Cement – Part 1: Composition, specifications and conformity criteria for common cements (Polish Committee for Standardization (PKN), 2012).

[30] PN-EN 196. -3:2006. Cement – Testing methods – Part 3: Determination of setting times and volume constancy (Polish Committee for Standardization (PKN), 2006).

[31] PN-EN 196-6:2011. Cement testing methods – Part 6: Determination of fineness of grinding (Polish Committee for Standardization (PKN), 2011).

[32] PN-EN 196-. 2:2013-11. Cement testing methods – Part 2: Chemical analysis of cement (Polish Committee for Standardization (PKN), 2013).

[33] 196-1:2016-07. Cement test methods – Part 1: Determination of strength (Polish Committee for Standardization (PKN), 2016).

[34] PN-EN 12350-9:2011. Testing fresh concrete – Part 9: V-funnel test (Polish Committee for Standardization (PKN), 2011).

[35] EFNARC. The European Guidelines for Self-Compacting Concrete: Specification, Production and Use. (2005).

[36] PN-EN 12350-10:2011. Testing fresh concrete – Part 10: L-box test (Polish Committee for Standardization (PKN), 2011).

[37] PN-EN 12350-12:2012. Testing fresh concrete – Part 12: J-Ring test (Polish Committee for Standardization (PKN), 2012).

[38] PN-EN 12390-. 3:2019-07. Concrete testing – Part 3: Compressive strength of test specimens (Polish Committee for Standardization (PKN), 2019).

[39] PN-EN 12390-13. :2021-12. Concrete tests – Part 13: Determination of secant compressive modulus of elasticity (Polish Committee for Standardization (PKN), 2021).

[40] PN-EN 12390-6:2011. Splitting tensile strength of test specimens (Polish Committee for Standardization (PKN), 2011).

[41] PN-EN 12390-. 5:2019-08. Testing hardened concrete – Part 5: Flexural strength of test specimens. Testing Hardened Concrete (Polish Committee for Standardization (PKN), 2019).

[42] RILEM TC 50-FMC. : Fracture mechanics test methods for concrete – Determination of fracture energy *Gf*. (1985).

[43] RILEM TC 89-FMT. : Determination of fracture parameters (*KIc* and *Gf*) – Size-effect method. (1990).

[44] Song, W. & Yin, J. Hybrid effect evaluation of steel fiber and carbon fiber on the performance of the fiber reinforced concrete. *Materials* **9**, 704 (2016). 28773824 10.3390/ma9080704 PMC5512526

[45] Çelik, Z. & Bingöl, A. F. Effect of basalt, polypropylene and macro-synthetic fibres on workability and mechanical properties of self-compacting concrete. *Challenge J. Struct. Mech.* **5**35. (2019).

[46] Gencel, O., Brostow, W., Ozel, C. & Martınez-Barrera, G. Mechanical properties of self-compacting concrete reinforced with polypropylene fibres. *Mater. Res. Innov.* **15**, 216–225 (2011).

[47] Ghodousian, O., Garcia, R., Ghodousian, A. & Ayandeh, M. Properties of fibre-reinforced self-compacting concrete subjected to prolonged mixing: An experimental and fuzzy logic investigation. *J. Build. Pathol. Rehabil.*10.1007/s41024-023-00374-3 (2024).

[48] Diddi, P., Sharma, P., Srivastava, A., Madduru, S. R. & Reddy, E. Influence of blends of alkali resistant fibres and polypropylene fibres on fresh and hardened properties of early strength self compacting concrete. *J. Build. Pathol. Rehabil.*10.1007/s41024-022-00219-5 (2022).

[49] Aslani, F. & Nejadi, S. Mechanical characteristics of self-compacting concrete with and without fibres. *Mag. Concr. Res.* **65**, 608–622 (2013).

[50] Ponikiewski, T. & Katzer, J. Fresh mix characteristics of self-compacting concrete reinforced by fibre fresh mix characteristics of self- compacting concrete reinforced by fibre. *Periodica Polytech. Civil Eng.* **61**(2), 226–231 (2016).

[51] Abousnina, R. et al. Mechanical properties of macro polypropylene fibre-reinforced concrete. *Polymers* **13**, 4112 (2021). 34883614 10.3390/polym13234112 PMC8659559

[52] Deng, Z. et al. A study of tensile and compressive properties of hybrid basalt-polypropylene fiber‐reinforced concrete under uniaxial loads. *Struct. Concr.*10.1002/suco.202000006 (2020).

[53] Fu, Q., Xu, W., Mengxin, B., Guo, B. & Niu, D. Effect and action mechanism of fibers on mechanical behavior of hybrid basalt-polypropylene fiber-reinforced concrete. *Structures* **34**, 3596–3610 (2021).

[54] Ayub, T., Shafiq, N. & Nuruddin, M. Mechanical properties of high-performance concrete reinforced with basalt fibers. *Procedia Eng.* **77**, 131–139 (2014).

[55] He, W., Kong, X., Ying, F., Zhou, C. & Zheng, Z. Experimental investigation on the mechanical properties and microstructure of hybrid fiber reinforced recycled aggregate concrete. *Constr. Build. Mater.* **261**, 120488 (2020).

[56] Smarzewski, P. Comparative fracture properties of four fibre reinforced high performance cementitious composites. *Materials* **13**, 2612 (2020). 32521702 10.3390/ma13112612 PMC7321617

[57] Liang, N. et al. Study on the fracture toughness of polypropylene–basalt fiber-reinforced concrete. *Int. J. Concr. Struct. Mater.*10.1186/s40069-021-00472-x (2021).

[58] ASTM C1609/C1609M-19. Standard test method for flexural performance of fiber-reinforced concrete (using beam with third-point loading) (ASTM International, 2019).

[59] Li, V. C. et al. Toughening mechanisms in cement-based composites. *Cem. Concr. Compos.* **14** (3), 143–157 (1992).

[60] Mohammadizadeh, S., Dalfre Filho, J. G., Trautwein, L. M. & Buttignol, T. E. T. Effect of steel and PVA microfibers on UHPFRC: Mechanical performance and microstructural analysis. *Case Stud. Constr. Mater.* **23**, e05295. 10.1016/j.cscm.2025.e05295 (2025).

[61] Al-Baghdadi, H. M. & Kadhum, M. M. Effects of different fiber dosages of PVA and glass fibers on the interfacial properties of lightweight concrete with engineered cementitious composite. *Buildings* **14**(8), 2379. 10.3390/buildings14082379 (2024).

[62] Soe, K., Zhang, Y. X. & Zhang, L. Material properties of a new hybrid fibre-reinforced engineered cementitious composite. *Constr. Build. Mater.* **43**, 399–407 (2013).

[63] Arslan, M. Effects of basalt and glass chopped fibers addition on fracture energy and mechanical properties of ordinary concrete: CMOD measurement. *Constr. Build. Mater.* **114**, 383–391 (2016).

[64] Wang, R., Wu, C., Yu, R. & Zhang, Z. Synergistic toughening mechanism of multi-scale fibres in ultra-high-performance concrete: Experimental and micromechanical modelling. *Constr. Build. Mater.* **407**, 133319 (2024).

